# Thromboinflammatory changes in plasma proteome of pregnant women with PCOS detected by quantitative label-free proteomics

**DOI:** 10.1038/s41598-019-54067-4

**Published:** 2019-11-26

**Authors:** R. K. Arffman, M. Saraswat, S. Joenväärä, M. Khatun, R. Agarwal, T. Tohmola, I. Sundström-Poromaa, R. Renkonen, T. T. Piltonen

**Affiliations:** 1 Department of Obstetrics and Gynecology, PEDEGO Research Unit, Medical Research Center, Oulu University Hospital, University of Oulu, Oulu, Finland; 2 Transplantation Laboratory, Haartman Institute, University of Helsinki, Helsinki, Finland; 3 HUSLAB, Helsinki University Hospital, Helsinki, Finland; 4 Department of Reproductive biology, All India Institute of Medical Sciences, Ansari Nagar, New Delhi, 110029 India; 5 Department for Women’s and Children’s Health, Uppsala University, Uppsala, Sweden

**Keywords:** Diagnostic markers, Mass spectrometry, Endocrine reproductive disorders

## Abstract

Polycystic ovary syndrome (PCOS) is the most common endocrinological disorder of fertile-aged women. Several adverse pregnancy outcomes and abnormalities of the placenta have been associated with PCOS. By using quantitative label-free proteomics we investigated whether changes in the plasma proteome of pregnant women with PCOS could elucidate the mechanisms behind the pathologies observed in PCOS pregnancies. A total of 169 proteins with ≥2 unique peptides were detected to be differentially expressed between women with PCOS (n = 7) and matched controls (n = 20) at term of pregnancy, out of which 35 were significant (p-value < 0.05). A pathway analysis revealed that networks related to humoral immune responses, inflammatory responses, cardiovascular disease and cellular growth and proliferation were affected by PCOS. Classification of cases and controls was carried out using principal component analysis, orthogonal projections on latent structure-discriminant analysis (OPLS-DA), hierarchical clustering, self-organising maps and ROC-curve analysis. The most significantly enriched proteins in PCOS were properdin and insulin-like growth factor II. In the dataset, properdin had the best predictive accuracy for PCOS (AUC = 1). Additionally, properdin abundances correlated with AMH levels in pregnant women.

## Introduction

Polycystic ovary syndrome (PCOS) is a complex, heterogeneous and often underdiagnosed endocrine disorder. According to the International PCOS Guideline, the syndrome can be diagnosed if at least two of three of the following criteria are fulfilled after exclusion of other etiologies: oligo- or anovulation, clinical and/or biochemical hyperandrogenism and polycystic ovaries^1^. The estimated prevalence varies from 8–12% depending on the study population and applied criteria^2–4^. As PCOS has a strong metabolic and inflammatory side, it should be considered more than a mere gynaecological problem. Indeed, affected women are at increased risk for metabolic syndrome, type II diabetes and cardiovascular diseases^5^. Moreover, low-grade chronic inflammation is commonly detected in women with PCOS, and it has been linked to the development of insulin resistance and accelerated atherosclerosis^6^.

Due to the vast scientific interest in PCOS, it has become evident that affected women also present with high pregnancy- related morbidity and adverse offspring outcomes^7,8^. Affected women have a 3 −4- fold increase in the risk of pregnancy-induced hypertension and pre-eclampsia and a 2-fold higher risk for preterm delivery independent of BMI^9^. Structural alterations of placentae from women with PCOS have been reported, even in uncomplicated pregnancies, possibly indicating abnormal placentation and defective placental function^10,11^.

Proteomic technologies have been used to study PCOS-related alterations in protein expression in the plasma, ovarian tissue, follicular fluid, adipose tissue and T cells^12–16^. A list of proteomic biomarkers for PCOS has also been published, showing an association with networks related to the coagulation system, cell cycle regulation, metabolism, apoptosis, immune system/inflammation, cell signalling, oxidative stress, insulin, adipose tissue regulation, cholesterol and cell structure^17^. Interestingly, the proteomics biomarkers detected in women with PCOS overlap with those detected in women with pre-eclampsia, a disorder defined by hypertension and proteinuria, often related to placental dysfunction^18^. It is important to note that all the previous proteomic analyses in women with PCOS have been conducted using samples from non-pregnant women, and to date, no proteomics studies have been published for pregnant women with PCOS. Given that the women with PCOS are at risk for adverse gestational outcomes, a proteomic analysis during pregnancy could clarify the mechanisms leading to these conditions. In general, pregnancy can be considered a stress test as well as a window of opportunity to estimate health risks in women later in life^19^. For PCOS, it could be possible to identify the affected women, especially those at risk for adverse health outcomes later in life, by discerning the differences in circulating proteins.

By adopting a quantitative label-free proteomics approach, plasma proteomes of samples from uncomplicated term pregnancies of non-obese women with PCOS were compared with those of controls matched for age and BMI. A total of 169 proteins with two or more unique peptides were differentially expressed between cases and controls, from which 35 passed the cut-off Mann-Whitney p-value of 0.05. These proteins formed protein-protein interaction networks related to humoral immunity, inflammation and cardiovascular disease. The data were further analysed by the principal component analysis (PCA). To determine how well the proteomic analysis could classify the women with PCOS and controls and to identify possible biomarkers for PCOS, three parallel methods were used: orthogonal projections to latent structure-discriminant analysis (OPLS-DA), hierarchical clustering (HCA) and self-organizing maps (SOMs). We also found that the detected proteins correlated with several circulating hormones. It was also of interest to identify novel biomarkers that could be utilized in PCOS diagnostics. A ROC-curve analysis revealed that in the data set, complement factor properdin (properdin) was able to classify cases and controls with very high accuracy. Interestingly, properdin abundances correlated with AMH levels at the end of pregnancy.

## Results

### Metadata

**Figure 1:**
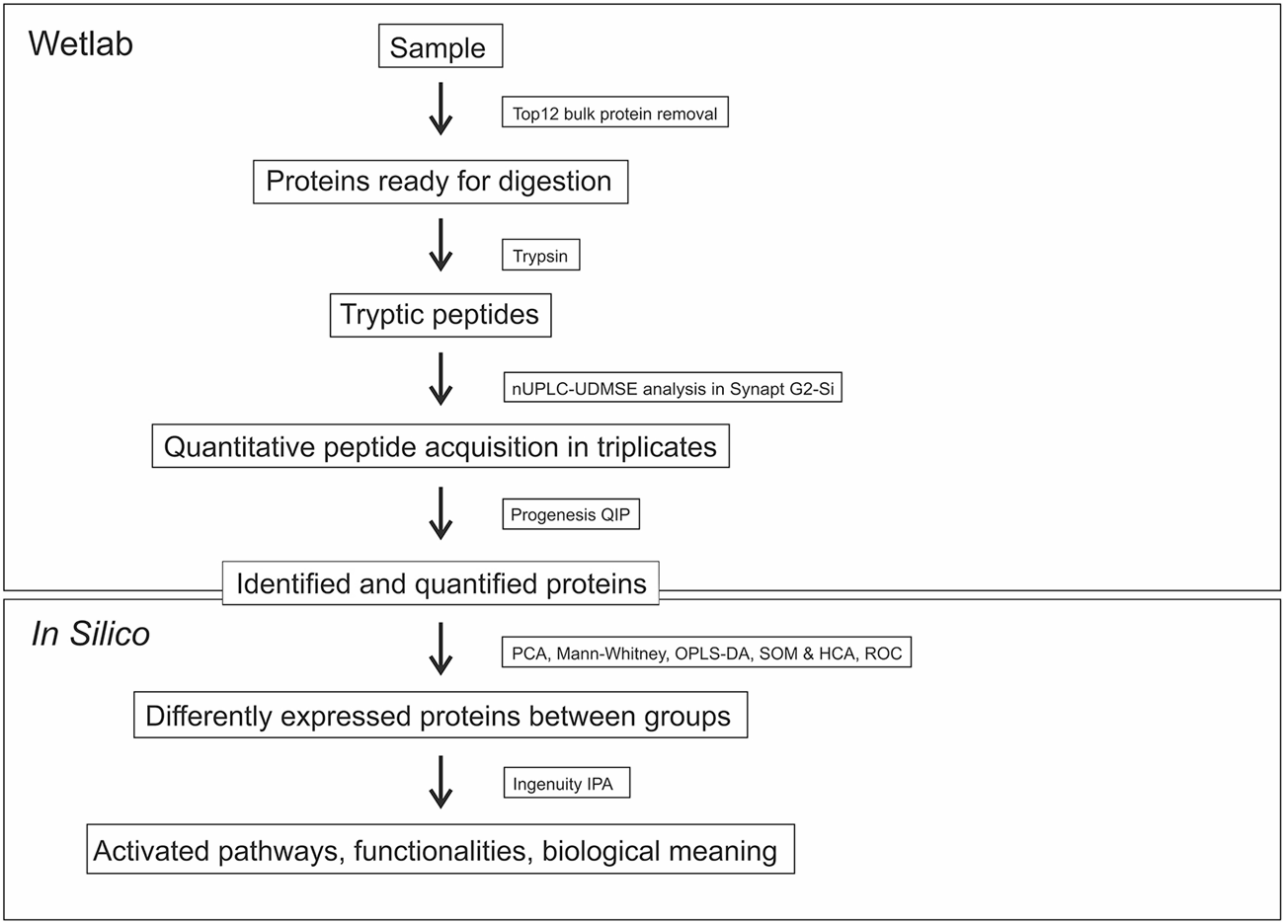
Quantitative Proteomics Analysis Workflow (see Methods for further information). Lithium-heparin plasma samples were depleted of the 12 most abundant proteins and digested with trypsin. Nanoflow ultrahigh performance liquid chromatography – Ultra-Definition Mass-spectrophotometry (nUPLC-UDMSE) was performed in triplicates. After acquisition, the data analysis was performed with Progenesis QI. Differences in protein abundances between groups were assessed by the Mann-Whitney U-test with a cut-off level set to p < 0.05. A network analysis by IPA was used to build protein interaction networks of proteins that differed between cases and controls and to provide a broader scope for interpretation regarding how the changes may affect the functions of the body. The Principal Component Analysis (PCA) was used to visualise the principal axes of protein abundance variations in cases and controls in order to define how much variation the sample classes has when compared and whether the cases and control separate from each others. A clustering analysis is an alternative technique to analyze the differences between groups as well as similarities within a group. Self-organizing maps (SOM) is an unsupervised data visualization technique that reduces the dimensions of data through the use of self-organising neural networks. Hierarchical clustering analysis (HCA) calculates the dissimilarity between individuals and builds a hierarchy of clusters. OPLS-DA was used to define differences between the groups and to identify the proteins with the highest discriminative power. These proteins were then used for the ROC-curve analysis to calculate AUC-values.

Label-free quantitative proteomics was performed on seven plasma samples from pregnant women with PCOS at term and on 20 plasma samples from pregnant control women matched for age and BMI. All plasma samples were collected at the delivery ward when the women arrived to give birth. A schematic representation of the entire analysis process is depicted in Fig. 1. Patient demographics are presented in Table 1. Furthermore, plasma Anti-Müllerian hormone (AMH) and steroid hormone analysis results from the authors’ previous study^20^ were added in the analyses as additional variables.

**Table 1:** Clinical characteristics of the study subjects. BMI, body-mass index; BP, blood pressure; SD, standard deviation; IQR, inter-quartile range; N, number of participants; AMH, Anti-Müllerian hormone.

Variable	PCOS (N = 7)		Control (N = 20)		p-value
	Mean/Median	SD/IQR	Mean/Median	SD/IQR	
Age, years	32	4.7	32	3.8	0.802
Pre-pregnancy BMI, kg/m^2^	21.79	3.98	23.36	3.50	0.310
Gestational weight gain (kg)	11.0	4.3	12.5	3.1	0.497
Gestational length,days	279	11	286	6	0.045*
Systolic BP, mmHg	123	11	125	10	0.7567
Diastolic BP, mmHg	76	7	77	6	0.871
Birthweight, g	3430	421	3863	573	0.161
Cesarean section (%)	14,3	—	25	—	0.656
AMH (ng/mL)	1.60	0.93–3.54	0.91	0.49–1.14	0.013*
Testosterone (nmol/L)	3.48	2.77–5.08	2.54	1.68–3.74	0.166
Estradiol (nmol/L)	80.59	17.76	71.35	39.7	0.302

### Proteomics in cases and controls

A total of 169 proteins with two or more unique peptides were identified, from which 35 passed the cut-off Mann-Whitney p-value of 0.05. The fold-changes of the proteins with a p-value < 0.05 ranged from 13,3 to −3.81. The proteins with two or more unique peptides detected and a Mann-Whitney p-value < 0.05 as well as their mean abundances are listed in Table 2. The exact standardized protein abundances for each individual are listed in Supplementary Table 1.

**Table 2:** List of proteins that were differentially abundant (p < 0.05) in the plasma of pregnant women at term with PCOS compared with age and BMI matched controls.

Protein name	UniProt ID	Peptide count	Unique peptides	Mann- Whitney P-value	Max fold change	Normalized abundance		Raw abundance	
						Control (mean ± SD)	PCOS (mean ± SD)	Control (mean ± SD)	PCOS (mean ± SD)
Properdin (CFP)	P27918	4	3	6,02E-05	13,3	9871 ± 8885	131111 ± 60591	8907 ± 7368	81473 ± 41346
Actin_ cytoplasmic 1 (ACTB)	P60709;P63261	12	3	6,91E-03	3,1	2019 ± 1857	6287 ± 6080	1747 ± 1199	3588 ± 2964
Insulin-like growth factor II (IGF2)	P01344	2	2	4,97E-04	2,6	38381 ± 23687	100387 ± 34863	34863 ± 17388	64047 ± 30921
Platelet factor 4 (PF4)	P02776;P10720	6	6	2,19E-03	2,0	96379 ± 83088	193262 ± 86578	89987 ± 54166	121068 ± 65809
F-box/LRR-repeat protein 6 (FBXL6)	Q8N531	2	2	3,64E-03	1,8	163796 ± 81585	297700 ± 135684	151970 ± 69540	192129 ± 120097
Protein SAA2-SAA4 (SAA2-SAA4)	A0A096LPE2;P35542;P0DJI9	11	8	1,84E-03	1,8	94904 ± 30973	166255 ± 61245	88176 ± 28302	106182 ± 50352
Platelet basic protein (PPBP)	P02775	8	7	9,35E-03	1,6	126680 ± 61697	207258 ± 69378	114575 ± 40132	129752 ± 60820
Coagulation factor XII (F12)	P00748	15	11	4,97E-04	1,6	168494 ± 49967	266963 ± 58132	164621 ± 67246	163966 ± 46991
Fibulin-1 (FBLN1)	P23142	24	19	6,91E-03	1,5	186638 ± 76342	283480 ± 90281	177325 ± 73434	178048 ± 73724
Thrombospondin-1 (THBS1)	P07996	9	6	6,91E-03	1,5	40101 ± 11892	60276 ± 25891	38058 ± 12812	41024 ± 30970
Ensconsin (MAP7)	Q14244	3	3	2,17E-02	1,4	140935 ± 43737	191963 ± 55106	134780 ± 49697	119405 ± 44316
Clusterin (CLU)	P10909	41	34	1,28E-03	1,4	104524 ± 182056	1411565 ± 233890	996799 ± 265839	899511 ± 341864
Complement factor H-related protein 4 (CFHR4)	Q92496	2	2	4,07E-02	1,3	6025 ± 3254	8120 ± 3551	5561 ± 2350	5138 ± 2453
Apolipoprotein A-IV (APOA4)	P06727	56	54	2,48E-02	1,3	1342773 ± 378828	1787655 ± 541403	1259649 ± 367488	1165558 ± 577026
Apolipoprotein C-III (APOC3)	P02656	26	23	2,48E-02	1,3	779875 ± 355305	1019474 ± 264894	739388 ± 350789	627452 ± 207791
Alpha-1B-glycoprotein (A1BG)	P04217	94	83	2,17E-02	1,3	5953838 ± 1342139	7716950 ± 1858329	5646416 ± 1498040	4962091 ± 2219956
Inter-alpha-trypsin inhibitor heavy chain H2 (ITIH2)	P19823	94	82	3,64E-03	1,3	3229720 ± 548599	4100130 ± 965612	3087860 ± 821714	2685395 ± 1383517
Hemopexin (HPX)	P02790;Q2M389Q8N987;Q9NZ08	122	111	1,44E-02	1,3	7363912 ± 1596736	9221228 ± 2118666	7115877 ± 2417683	5933958 ± 2625698
Inter-alpha-trypsin inhibitor heavy chain H1 (ITIH1)	P19827	79	70	3,19E-02	1,2	3496796 ± 808572	4264955 ± 1024208	3322647 ± 963914	2795958 ± 1499041
Apolipoprotein E (APOE)	P02649	48	45	2,48E-02	1,2	1265005 ± 264966	1521778 ± 301299	1207847 ± 350338	960169 ± 347148
Apolipoprotein C-IV (APOC4)	P55056	9	8	2,48E-02	1,2	89509 ± 50207	107081 ± 28316	81235 ± 31210	66879 ± 24291
Plasminogen (PLG)	P00747;Q15195;Q02325;P35900	103	93	3,19E-02	1,2	3257229 ± 949755	3823210 ± 676537	3098101 ± 1041808	2449869 ± 996846
Vitronectin (VTN)	P04004	79	62	3,19E-02	1,2	2833870 ± 532068	3299916 ± 652184	2721551 ± 767587	2111094 ± 875460
Complement component C8 alpha chain (C8A)	P07357	29	25	4,58E-02	−1,2	471877 ± 74769	409700 ± 99288	458636 ± 142589	266387 ± 142378
Complement component C8 beta chain (C8B)	P07358	30	25	3,61E-02	−1,2	673193 ± 146055	574634 ± 243926	649089 ± 200788	389647 ± 289951
Carboxypeptidase B2 (CPB2)	Q96IY4	13	11	1,66E-02	−1,2	210142 ± 48905	174108 ± 43031	200478 ± 61439	114195 ± 61836
Serum paraoxonase/arylesterase 1 (PON1)	P27169	27	23	2,81E-02	−1,3	1370720 ± 411430	1070315 ± 482501	1335219 ± 518580	722929 ± 570982
Zinc-alpha-2-glycoprotein (AZGP1)	P25311	18	16	4,07E-02	−1,3	262328 ± 64339	199592 ± 56997	256660 ± 92476	129727 ± 64001
Prostaglandin-H2 D-isomerase (PTGDS)	P41222	3	2	4,29E-03	−1,5	7234 ± 1724	4701 ± 1943	6932 ± 2207	2910 ± 1249
Glyceraldehyde-3-phosphate dehydrogenase (GAPDH)	P04406	4	4	4,58E-02	−1,8	12975 ± 14276	7261 ± 2383	11191 ± 8804	4770 ± 3123
Complement factor H-related protein 5 (CFHR5)	Q9BXR6	4	2	2,81E-02	−1,8	9172 ± 4751	5084 ± 2454	9080 ± 5239	3449 ± 2385
Cystatin-C (CST3)	P01034	3	2	5,04E-03	−1,9	24739 ± 10617	13244 ± 4178	24523 ± 13612	8542 ± 4398
Granzyme M (GZMM)	P51124	2	2	2,19E-03	−3,3	171820 ± 102607	52349 ± 80914	165486 ± 97526	41860 ± 75989
Embryonic growth/differentiation factor 1 (GDF1)	P27539	2	2	2,17E-02	−3,8	35570 ± 90017	9334 ± 6358	27475 ± 55416	6343 ± 6445

### Pathway analysis

The Ingenuity pathway analysis (IPA) network module was used to identify the protein interaction networks of proteins differentially expressed in pregnant women with PCOS compared to pregnant control women. Only the proteins passing the cut-off p-value of 0.05 from the Mann-Whitney analysis were used. Network analysis reveals shared functional and biological aspects between the proteins which makes it easier to interpret how the proteomic changes affect the body as a whole. The networks that were identified were related to humoral immune responses, inflammatory responses, cardiovascular disease and cellular growth and proliferation. Full lists of proteins in these two networks can be found in Supplementary Tables 2 and 3.

### Principal component analysis (PCA)

**Figure 2:**
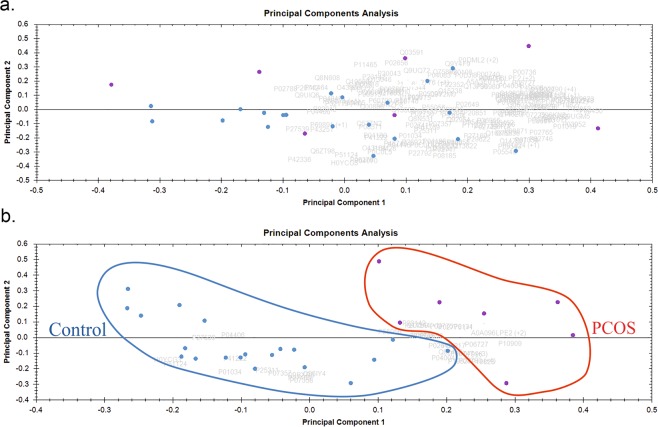
Principal component analysis. Purple dots represent PCOS cases (circled in red) and blue dots the controls (circled in blue). (**a**) All differentially expressed proteins with ≥2 unique peptides are presented (**b**) Only proteins passing the cut-off of p < 0.05 for Mann-Whitney test are depicted. The cases and controls mostly cluster separately along the x-axis.

A PCA was done using Progenesis QI Proteomics to determine the principal axes on protein abundance variations in PCOS cases and controls. PCA reduces the data to its basic components so that patterns and clusters can be detected. The analysis with all the proteins with two or more unique peptides did not show distinct clustering (Fig. 2b), however, when PCA was carried out with only the proteins with two or more unique peptides and a Mann-Whitney p-value < 0.05 the cases and controls mostly separated along the X-axis (Fig. 2a), revealing that the proteomes of pregnant women with PCOS resemble each others.

### Hierarchical clustering and SOM clustering

**Figure 3:**
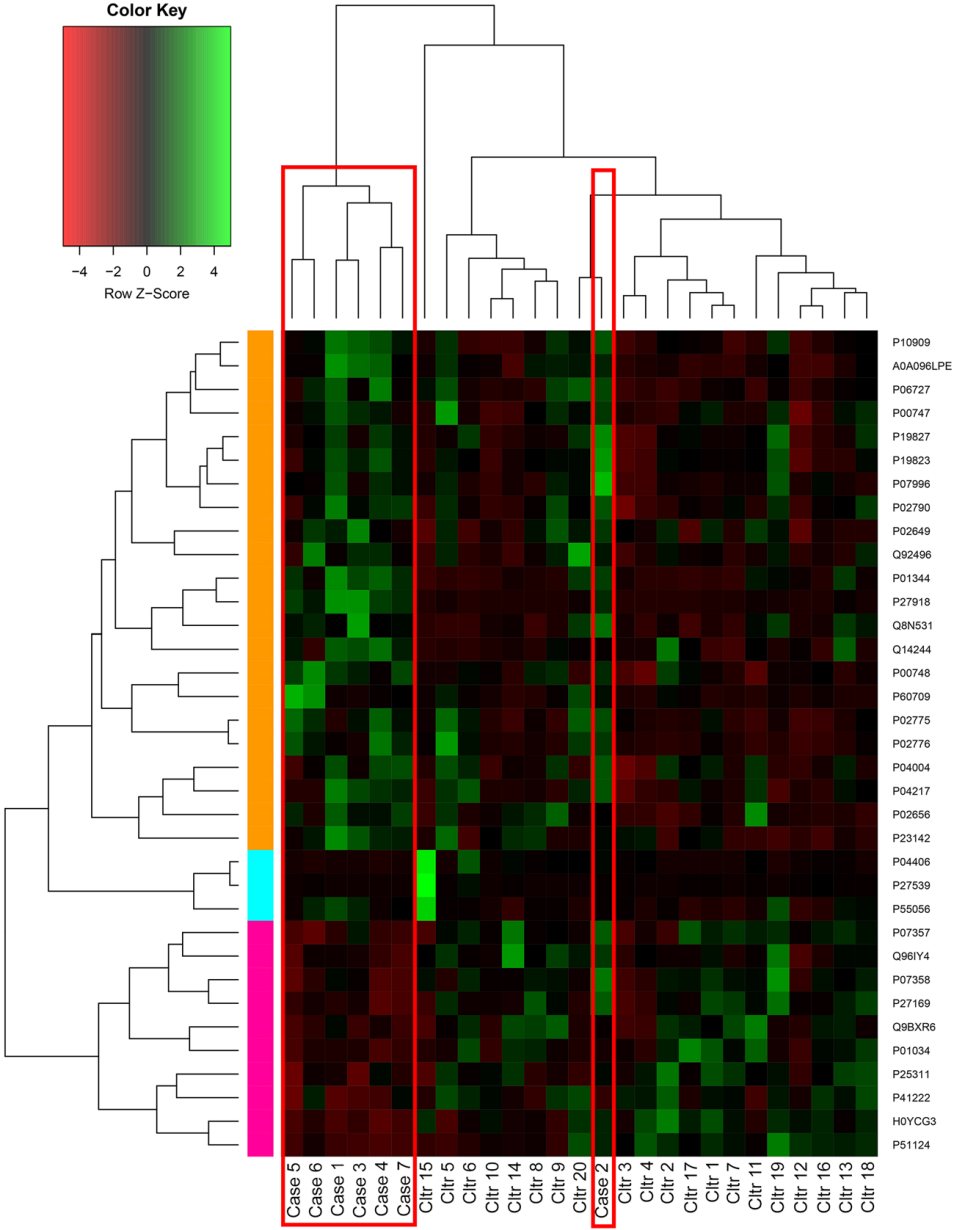
Hierarchical clustering analysis (HCA) of the 35 differentially expressed proteins in cases and controls. 5/6 of the cases clustered together.

**Figure 4:**
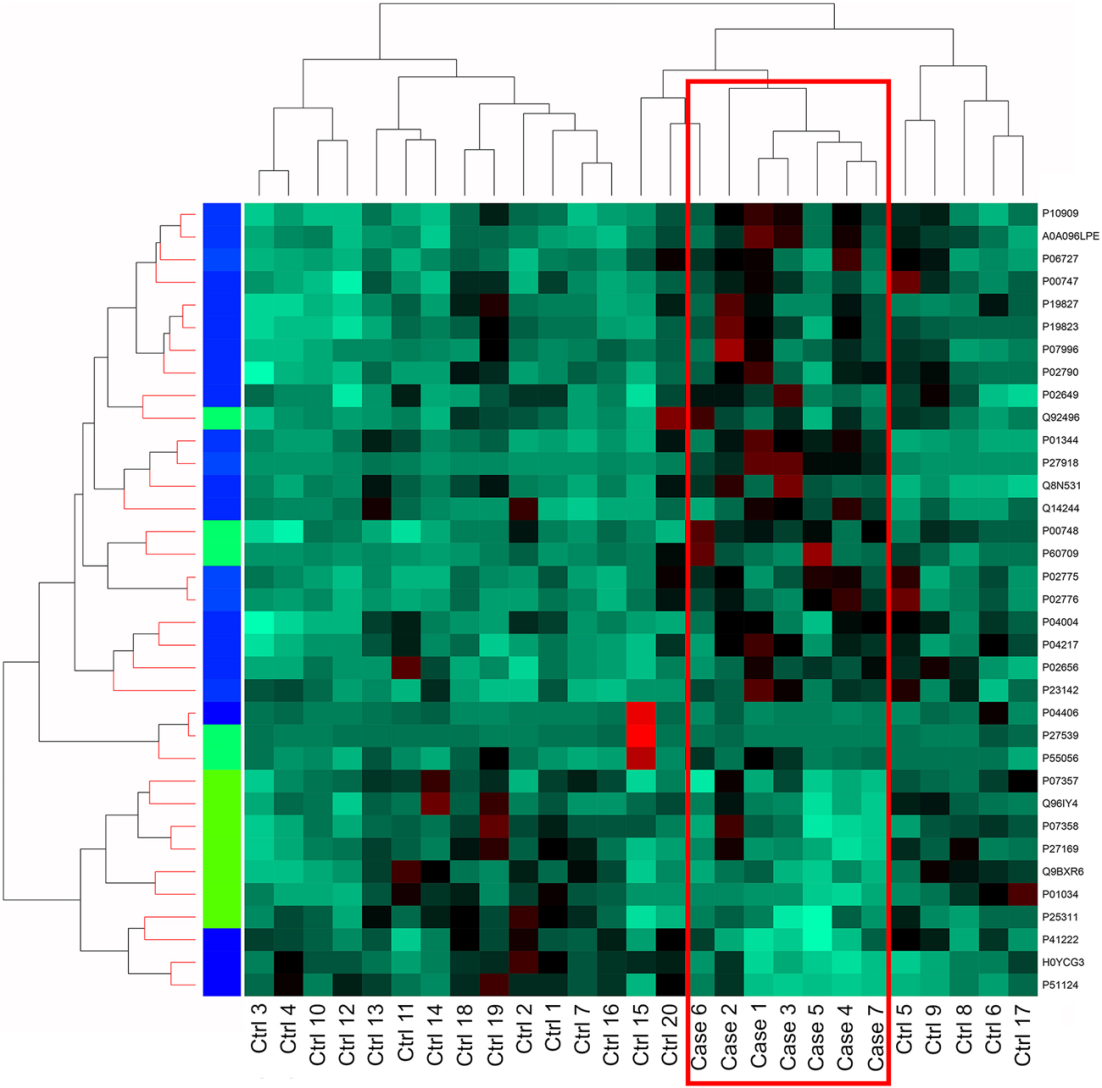
Self-organizing map (SOM) clustering of the 35 differentially expressed proteins in cases and controls. All cases clustered together.

Protein abundance data of PCOS vs control (top 35 Mann-Whitney Passing proteins only) were used for hierarchical clustering (HCA) and self-organizing map (SOM) clustering analyses to determine which samples cluster together. Clustering analyses are unsupervised methods, so the only information provided is the protein abundance data, based on which the process clusters the individuals whose proteomes most resemble each other. In hierarchical clustering analysis 6/7 cases of PCOS clustered together (Fig. 3). In SOM clustering, however, all cases cluster next to each other (Fig. 4). This shows that based on only the protein abundances, unsupervised methods find enough similarities in proteomes of pregnant women with PCOS to cluster them together.

### Orthogonal projections to latent structure-discriminant analysis (OPLS-DA)

OPLS-DA modelling was used to identify proteins that can differentiate the cases from controls. OPLS-DA is a modelling technique that can perform binary comparisons. The modelling provides two values: (p) is the magnitude of change of a given marker, and (p(corr)) depicts significance of the marker in binary comparison. Any experiment with a large number of measured variables can be modelled with OPLS-DA to filter out the most differing markers among the two groups. OPLS-DA can separate predictive and uncorrelated variance in binary comparisons. According to OPLS-DA, two proteins that passed the cutoff value of +0.65 or −0.65 for p(corr) were enriched in the plasma of pregnant women with PCOS: properdin (CFP) (p(corr) value = −0.850) and insulin-like growth factor II (IGF-II) (p(corr) = −0.691), indicating them as potential discriminant markers in our dataset.

### ROC curve analysis

**Figure 5:**
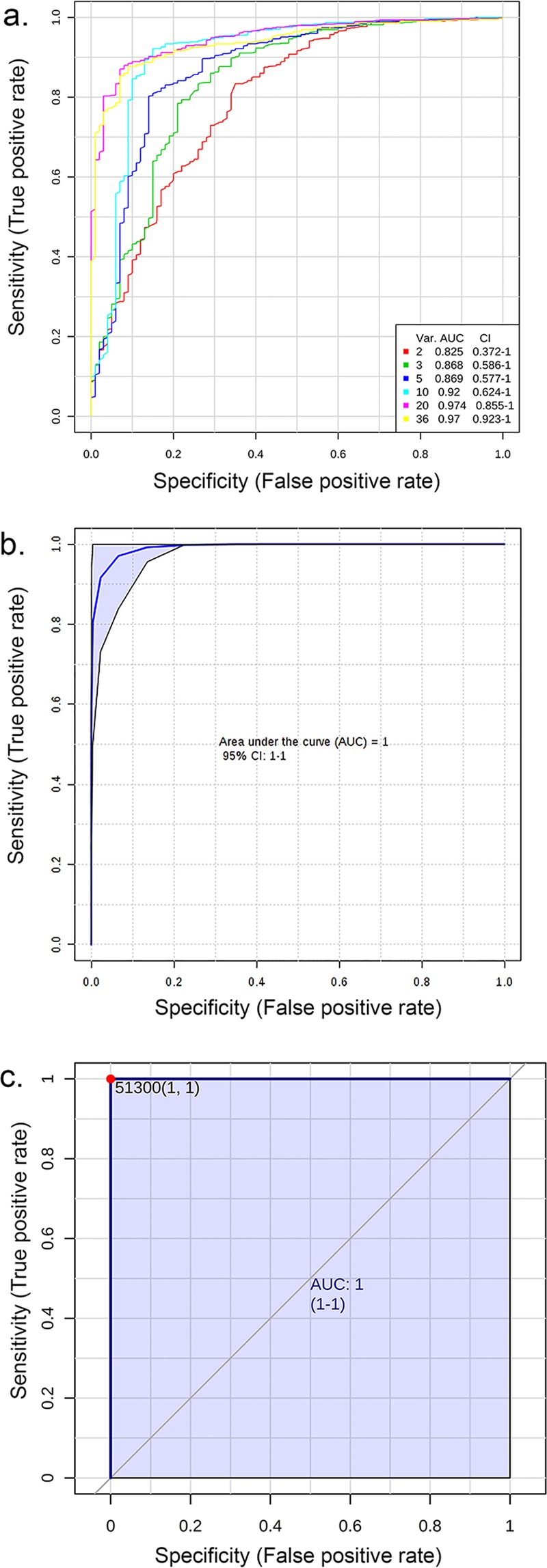
ROC-curve analysis using individual or a combination of proteins. (**a**) Combination ROC-curves calculated by MetaboAnalyst 4.0. The best AUC-value was reached using all the identified 35 proteins and AMH (yellow, AUC: 0.97). See Suppl. Table 4 for proteins used for creation of the curves. (**b**) AUC-curve of IGF-2 AUC: 0.937. c. An AUC-curve of properdin alone can classify cases and controls perfectly, AUC: 1.

To identify the proteins that could be used to distinguish the cases from controls, a ROC curve analysis was performed using Metaboanalyst analysis tool^21^. The top 35 plasma proteins and AMH-levels were used for calculating ROC curves for individual markers as well as combination ROC curves for sets of biomarkers by a support vector machine algorithm (Fig. 5a and Suppl. Table 4). Properdin alone had the best predictive accuracy (AUC-value = 1, Fig. 5c) but IGF-2 also performed well (Fig. 5b). Of note, properdin was also the top protein found by OPLS-DA in the dataset. This serves as the orthogonal statistical validation of the OPLS-DA modelling and non-parametric testing.

### Correlation analysis

In order to see which clinical parameters correlated with protein abundances measured with LC-MS, we used clinical data as well as steroid hormone and AMH values form our previous study^20^. All significant correlations are listed in Table 3. Interestingly, properdin levels correlated positively with AMH levels (r = 0.417, p = 0.0383). As AMH can be considered a marker for antral follicle count, high properdin levels may be linked with polycystic ovarian morphology. The strongest correlation was detected between inactive dipeptyl peptidase 10 (DPP10) and testosterone (r = 0.700, p < 0.001).

**Table 3:** Correlation analysis results for clinical parameters and protein abundances detected by LC-MS. Presented as correlation coefficient (p-value).

Protein	Accession	AMH	Androstenedione	Testosterone	Progesterone	Estradiol	Estriol	Estrone	Estrone-3-sulphate
Granzyme M	P51124	−0.574 (0.0027)	−0.444 (0.0262)		−0.424 (0.0349)				
Unknown	H0YCG3	−0.541 (0.0053)	−0.505 (0.0101)		−0.486 (0.0138)	−0.500 (0.0109)			
Unknown	H0YJW9	−0.498 (0.0113)	−0.589 (0.0020)		−0.483 (0.0144)	−0.403 (0.0455)			
Complement component C8 gamma chain	P07360	−0.431 (0.0317)	−0.530 (0.0065)			−0.436 (0.0292)	−0.403 (0.0455)		
Properdin	P27918	0.417 (0.0383)							
Serum amyloid P	P02743	−0.405 (0.0443)							
Fanconi associated nuclease	Q9Y2M0	−0.404 (0.0452)							
Heparin cofactor 2	P05546	−0.402 (0.0463)			−0.410 (0.0416)	−0.430 (0.0319)			
Fetuin-B	Q9UGM5	−0.397 (0.0493)	−0.404 (0.0451)		−0.435 (0.0298)				
C4b-binding protein alpha chain	P04003		−0.583 (0.0022)						
Apolipoprotein C-III	P02656		0.525 (0.0071)	0.645 (4.96E-04)	0.430 (0.0319)	0.507 (0.0097)			
Insulin-like growth factor-binding protein complex acid labile subunit	P35858		−0.523 (0.0073)		−0.459 (0.0209)				
Ficolin-3	O75636		−0.466 (0.0190)		−0.442 (0.0270)				
Keratin type I cytoskeletal 9	P35527		−0.434 (0.0304)		−0.526 (0.0070)				
Phosphatidylinositol 4.5-bisphosphate 3-kinase catalytic subunit alpha	P42336		−0.430 (0.0317)						
Prostaglandin-H2 D-isomerase	P41222		−0.422 (0.0357)		−0.399 (0.0483)				
Pregnancy-specific beta-1-glycoprotein 3	Q16557		−0.417 (0.0379)				−0.513 (0.0088)		
Kallistatin	P29622		−0.416 (0.0384)						
Sex hormone-binding globulin	P04278		−0.416 (0.0388)				−0.406 (0.0442)		
Tetranectin	P05452		−0.413 (0.0402)		−0.555 (0.0040)	−0.412 (0.0407)	−0.424 (0.0347)		
Complement C3	P01024;Q96MT0		−0.411 (0.0415)						
Zinc-alpha-2-glycoprotein	P25311		−0.400 (0.0474)		−0.477 (0.0158)				
Inactive dipeptidyl peptidase 10	Q8N608			0.700 (9.96E-05)		0.646 (0.0005)		0.479 (0.0155)	
Attractin	O75882			0.566 (3.17E-03)					
Inter-alpha-trypsin inhibitor heavy chain H4	Q14624			0.560 (3.62E-03)					
Complement factor H-related protein 5	Q9BXR6			0.404 (4.52E-02)					
Pappalysin-1	Q13219				0.568 (0.0031)				
Platelet factor 4	P02766				−0.530 (0.0064)				
Vitamin K-dependent protein C	P04070				−0.460 (0.0207)	−0.430 (0.0318)			
Vitamin D-binding protein	P02774;Q5VZM2				−0.448 (0.0248)				
HLA class II histocompatibility antigen DP beta 1 chain	P04440				−0.447 (0.0252)				
Extracellular matrix protein 1	Q16610				−0.438 (0.0286)				
Ceruloplasmin	P00450				−0.437 (0.0289)				
Beta-Ala-His dipeptidase	Q96KN2				−0.425 (0.0341)				
Keratin type II cytoskeletal 1	P04264				−0.409 (0.0422)				
Thyroxine-binding globulin	P05543				−0.574 (0.0027)	−0.509 (0.0093)		−0.463 (0.0197)	
Pregnancy-specific beta-1-glycoprotein 9	Q00887					0.483 (0.0145)	0.475 (0.0164)		
ADP-ribosyl cyclase/cyclic ADP-ribose hydrolase 2	Q10588					0.433 (0.0308)		0.588 (0.0020)	
Complement component C8 beta chain	P07358					−0.411 (0.0414)			
Complement C1r subcomponent-like protein	Q9NZP8						0.440 (0.0278)		
Actin cytoplasmic 1	P60709;P63261						0.406 (0.0442)		
Alpha-1-antichymotrypsin	P01011							−0.463 (0.0198)	
Pregnancy-specific beta-1-glycoprotein 1	P11464							0.449 (0.0242)	
Pregnancy-specific beta-1-glycoprotein 5	Q15238								0.477 (0.0158)

## Discussion

PCOS affects around 8–12% of the female population, making it one of the most common endocrinological disorders worldwide^2–4^. Although the affected women suffer from reproductive and metabolic dysfunction, the syndrome often remains undiagnosed^22^. PCOS is a risk factor for adverse pregnancy outcomes, such as pregnancy induced hypertension, pre-eclampsia, prematurity and gestational diabetes, but the underlying mechanisms remain unclear^7,9^. During pregnancy major metabolic and inflammatory changes occur in the female body. To determine whether these responses are affected by PCOS, plasma samples from uncomplicated term pregnancies from non-obese women with PCOS and matched controls were compared using label-free quantitative proteomics. To the authors’ knowledge, this is the first study to assess the plasma proteome of pregnant women with PCOS.

The analysis indicated that 35 proteins were significantly differentially expressed between the cases and the controls. Most of the proteins were associated with networks related to inflammation, humoral immunity and cardiovascular disease. Amongst these were some proteins previously detected in non-pregnant women with PCOS, but proteins that have not been associated with PCOS previously were also identified. SOM clustering and hierarchical clustering analyses revealed that the PCOS cases cluster close to each other. The support vector machine based ROC analysis was used to identify individual or combinations of proteins that could best classify cases from controls, and it revealed that properdin (CFP) alone provided the best prediction for PCOS diagnosis in thedataset.

Several of the proteins now identified in pregnant women with PCOS have been reported to be differentially expressed in non-pregnant women with PCOS as well, including increased levels of insulin growth factor II (IGF2), platelet factor 4 (PF4), serum amyloid A (SAA), fibulin-1 (FBL1), apolipoprotein A4 (APOA4) and alpha-1B-glycoprotein (A1BG) and decreased levels of zinc-alpha-2-glycoprotein (AZGP1) and serum paraoxonase/arylesterase 1 (PON1)^23–27^. In contrast to this finding, for non-pregnant women with PCOS, serum thrombospondin-1 (THBS1) levels have been reported to be lower^28^. Some of the proteins that were shown to be increased in the plasma of pregnant women with PCOS in the present study have been detected to be decreased in the follicular fluid of women with PCOS^14^. Interestingly, all these proteins are involved in thrombosis, inflammation and/or metabolism.

Indeed, the network analysis revealed that a vast majority of the differentially expressed proteins belonged to networks related to humoral responses, inflammatory responses, cardiovascular disease, lipid metabolism and cellular growth and proliferation. Even a normal pregnancy is an acquired hypercoagulable and inflammatory state^29,30^. The concentrations of coagulation factors increase and fibrinolysis and anticoagulatory factors decrease during gestation^29^. Furthermore, insulin sensitivity decreases by 50–60%^31^. All these changes are mandatory for the mother to adapt to pregnancy and on the other hand, a rigorous control of these factors is critical for a healthy pregnancy. Conditions that predispose to thrombosis, inflammation or insulin resistance may affect this fine balance, leading to suboptimal implantation and placentation and possibly complications during pregnancy.

The protein levels of IGF-2 were significantly higher in women with PCOS in our dataset. IGF-2 has been linked to placental function in several studies: *Igf2* overexpression in mice leads to overgrowth of both the placenta and the fetus^32^ and deletion of the placental-specific *Igf2* leads to reduction in placental and fetal weight and decreased transport of nutrients and reduced diffusion capacity^33,34^. In quinea pigs, administration of IGF-II to the mother in early-pregnancy increases placental functional capacity and weight of the fetus and the placenta^35^. Women with PCOS have a higher risk for large for large for gestational age (LGA) infants in general^8^, which could be partly explained with higher IGF-2 levels in their circulation. In our dataset, IGF-2 protein levels at the end of pregnancy did not correlate with fetal weight, however, we did not have any LGA infants in the PCOS group. Unfortunately, we did not have the information for placental weight to correlate with the IGF-2 levels.

Women with PCOS have a 3–4-fold higher risk for developing pre-eclampsia (PE) during pregnancy^9^. Pre-eclampsia is characterised by hypertension (≥140/90 mmHg) and proteinuria (>300 mg/day) after the 20th week of gestation. PE is assumed to stem from defective vascular function and placentation leading to hypoxia of the placenta, the release of soluble factors and over time, to generalised inflammation and progressive endothelial damage^36^. It has been reported that the proteomic markers of PCOS overlap with those identified in pre-eclamptic patients^18^, however, none of the PCOS studies utilised in the systematic review included data of pregnant women. Like PCOS, pre-eclampsia is a multi-systemic syndrome, where complex pathophysiological changes, including endothelial dysfunction, inflammation, activation of coagulation and metabolic changes are prominent features. Taking all this into account, it is not surprising that many of the proteins that were differentially expressed between pregnant PCOS cases and controls in the dataset have also been linked to pre-eclampsia, e.g. properdin, insulin-like growth factor 2 (IGF2), PF4, coagulation factor XII (F12), FBL1, apolipoprotein C3 (APOC3), hemopexin (HPX), apolipoprotein E (APOE), PLG, vitronectin (VTN), ZAG1, prostaglandin-H2 D-isomerase (PTGDS) and the complement component C8 alpha chain (C8A)^18^. It is interesting to note that these differences in circulating proteins were detected even though the women with PCOS included in our study had no signs of pre-eclampsia and a histological examination of their placentas revealed no abnormalities. Whether the presence of these markers can explain the increased risk of pregnancy complications or can be used to predict cardiometabolic risk later in life requires further research.

Complement factor properdin is a positive regulator of the alternative pathway but it can also act as an independent complement activator^37^. Properdin also seems to control platelet aggregation by exacerbating thromboinflammation^38^. In this dataset, properdin was enriched in the plasma of pregnant women with PCOS compared with controls. Disturbances in complement activation have been detected in women with PCOS previously, as complement factor C3 has been reported to correlate with traditional cardiovascular disease risk factors in these women, most importantly with insulin resistance^39,40^. Excessive complement activation during pregnancy has been linked to many pregnancy complications, such as pre-eclampsia, preterm birth and pregnancy-induced hypertension^41^. Properdin also had the highest p(corr)-value according to the OPLS-DA analysis, and the ROC-curve analysis showed that properdin alone could classify the cases and controls with high accuracy. Interestingly, properdin levels correlated with AMH levels, indicating a possible link between these two proteins. Unfortunately, the increase in properdin levels could not be confirmed using commercially available ELISAs (data not shown). This is most likely due to the fact that upon a freeze-thawing process properdin forms tight aggregates that resist even highly reducing conditions^38^. Due to the harsh sample reducing steps of LC/MS, it is well suited for the detection of properdin, unlike ELISA which preserves the structure of the antigen.

Correlation analysis of the identified protein abundances with clinical parameters, steroid hormones or AMH revealed several significant correlations. The most significant correlations were observed with testosterone, such as between testosterone and inactive dipeptidyl peptidase (DPP10; r = 0.700). DPP10 genes have previously been reported to be enriched in patients with autism^42^, a condition which incidence has been reported to be higher in women with PCOS and their offspring^43^. Androstenedione levels correlated negatively with sex-hormone binding globulin, cardiovascular protective factors (e.g. kallistatin^44^) and lipolytic factors (e.g. zinc-alpha-2-glycoprotein^45^), and both androstenedione and testosterone correlated with several complement factors, indicating that androgens affect plasma proteome of pregnant women.

In this study, data independent acquisition in the UDMSE mode analysis of clinical samples by LC-MS was used. Some of the reasons it was chosen it over ELISA- type orthogonal methods include the superior selectivity, reproducibility and multiplexing of mass spectrometric methods. Other reasons include higher throughput, cost-per-sample and sensitivity compared to immunoassays. However, due to very high dynamic range of serum/plasma proteome, it becomes necessary to deplete high-abundant proteins before processing samples for MS analysis. It introduces a confounding factor when considering validation by orthogonal methods due to the non-selective partial depletion of other proteins; however, current commercially available kits for high-abundant protein depletion are robust enough to maintain high inter-assay reproducibility. The same cannot be said for concordance between immunoassay and LC-MS assays. Regarding the statistical data analysis, reproducibility of selecting biomarkers for differentiating cases from controls is best achieved by a combination of unsupervised and supervised methods. A balanced combination of both of these method types in the form of the PCA, OPLS-DA and ROC curve analysis has been used. Whether a proteomic signature can separate the clinical groups in question is best determined using unsupervised methods, such as the PCA, but to identify important features of interest, supervised methods, such as the OPLS-DA are more suitable. Unsupervised methods serve as a guide regarding whether to use supervised methods or not because using supervised methods alone produces the risk of over-fitting the model.

In addition to the strengths, the study has some limitations. The pilot nature of the study is acknowledged as only seven women with PCOS were included in the analysis. Analyses of larger sample sets are thus warranted in the future. The cases were identified based on hospital records retrospectively and thus we had no contact with them before pregnancy. This population based approach does, however, also reduce selection bias, as the study population is not selected from patients from fertility clinics. The information collected during pregnancy was limited to the data collected from public health care and thus no data were available on insulin sensitivity of the subjects or weight of the placentas, for example. Placental samples were not available for *in vitro* studies and thus it was not possible to investigate how factors identified in this study affect placental function. In addition, all samples were from term uncomplicated pregnancies of non-obese women. Whether changes similar to the ones detected can be observed in obese women with PCOS or in samples obtained from early pregnancy requires further investigation.

Finally, it is hypothesized that the increased plasma levels of properdin and other circulating thromboinflammatory factors may indicate defective placental function or by themselves induce pathological changes that lead to increased incidence of pregnancy-related hypertensive disorders in women with PCOS. The finding that healthy pregnant women with PCOS display altered plasma proteome even during uncomplicated pregnancies may also reflect an increased risk for metabolic morbidity later in life. Future studies comparing systemic and placental expression of the biomarkers are essential for confirmation of these hypotheses.

## Methods

### Patients

The plasma samples were obtained from women who participated in the ‘Biology, Affect, Stress, Imaging, and Cognition in pregnancy and the puerperium’ (BASIC) cohort^20,30,46,47^. In Uppsala County, Sweden, all pregnant women are invited to participate in the population based BASIC cohort during their routine ultrasound at gestational week 16–18. The eligibility criteria are: 1.) ability to communicate in Swedish, 2.) age > 18 years and 3.) free from blood-borne diseases. The BASIC cohort covers around 23% of the women in the Uppsala county area. Brief demographic data are collected upon inclusion (e.g. on chronic disorders, ongoing medication, smoking in early pregnancy, height and weight). The samples used in this study were collected during years 2010–2012. The women with PCOS were identified from the cohort from the hospital register by the ICD-10 diagnosis of polycystic ovary syndrome (E282). The women were diagnosed according to the Rotterdam criteria, meaning that at least two of the following criteria were present: (1) polycystic ovaries by ultrasonography, (2) oligo- or amenorrhoea and (3) hyperandrogenism, either biochemical (elevated testosterone, androstenedione or elevated free androgen index) or clinical (hirsutism, Ferriman-Gallwey score > 8). The controls were healthy pregnant women matched for age and pre-pregnancy BMI and they had no records for PCOS diagnosis, menstrual irregularities or ovulatory infertility. Placental sample slides from the pregnancies were examined by a pathologist and no abnormalities were detected in either group.

The women provided written informed consent for inclusion and the study has been approved by the Regional Ethical Review Board (Uppsala, Sweden) and the Regional Ethical Committee of Northern Osthrobothnia Hospital District (Oulu, Finland). The study is compliant with the Declaration of Helsinki.

### Plasma sample collection

Upon admission to the delivery ward at the Uppsala county hospital, the only delivery ward in the area, a venous blood sample was drawn into a Lithium-Heparin tube. The plasma samples were stored at −70 °C.

### Plasma sample processing

Plasma samples were processed essentially as described previously^48,49^. Briefly, the top 12 most abundant proteins of plasma were depleted by a TOP 12 depletion kit (Pierce, Thermo Fisher) from 10 µL plasma according to the manufacturer’s instructions. Depleted plasma was used for estimating the protein amount by a BRADFORD MX reagent (EXPEDION) and an equal amount of protein per sample was dried and resuspended in 50 mM Tris buffer containing 6 M urea (pH 7.8). Dithiothreitol (DTT) was added to final concentration of 10 mM and sample tubes shaken for 1 h at RT. Iodoacetamide was then added to final concentration of 40 mM and tubes shaken for 1 h at RT. DTT (40 mM) was then used to quench excess IAA for 1 h at RT with shaking. Trypsin was the added to the protein mixtures at a trypsin:protein ratio of 1:50 and the samples were incubated at 37 °C overnight. Resulting tryptic peptides were cleaned with C18 spin columns according to the manufacturer’s instructions.

### UDMSE and data analysis

A total of 500 ng of peptides per samples were injected into the nano Acquity UPLC (Ultra Performance Liquid Chromatography) ‐ system (Waters Corporation, MA, USA). TRIZAIC nanoTile 85 μm × 100 mm HSS‐T3u wTRAP was used for the on-line liquid chromatographic separation of the peptide mixture before being analysed by a mass spectrometer. Samples were loaded, trapped and washed for 2 min with 8.0 μL/min with 1% B. The analytical gradient used was as follows: 0–1 min 1% B, at 2 min 5% B, at 65 min 30% B, at 78 min 50% B, at 80 min 85% B, at 83 min 85% B, at 84 min 1% B and at 90 min 1% B with 450 nL/min. Buffer A: 0.1% formic acid in water and Buffer B: 0.1% formic acid in acetonitrile.

Data were acquired in data independent acquisition mode using UDMSE with Synapt G2‐Si HDMS (Waters Corporation, MA, USA). The data were collected for 100–2000 m/z, with a scan time of one‐second, and a IMS wave velocity of 650 m/s, and collision energy was ramped in trap between 20 and 60 V. Calibration was performed using Glu1‐Fibrinopeptide B MS2 fragments and as a lock mass, the Leucine-Enkephaline ion (m/z 556.2771) was used during the runs. The samples were run as triplicates, and further analyses were done using Progenesis QI for Proteomics software (Nonlinear Dynamics, Newcastle, UK).

The data analysis was performed as previously described^48,49^. Briefly, the raw files were imported to Progenesis QI for Proteomics software (Nonlinear Dynamics, Newcastle, UK) using lock mass correction. Default parameters for peak picking and the alignment algorithm were used. Progenesis software facilitated peptide identification with Protein Lynx Global Server and label‐free quantification^50^. Peptide identification was done against Uniprot human FASTA sequences (UniprotKB Release 2017_03, 20183 sequence entries) with (CLPB_ECOLI (P63285)), and the ClpB protein sequence was inserted for label‐free quantification. The modifications used included fixed modification of cysteine (carbamidomethyl) and variable modification of methionine (oxidation). Trypsin was the digesting agent with two missed cleavages allowed. Fragment and peptide error tolerances were set to auto, and the FDR to less than 1%. One or more ion fragments per peptide, three or more fragments per protein and one or more peptides per protein were needed for ion matching.

The identified proteins were grouped based on the parsimony principle, and unique peptides to the protein are reported. The parsimony principle governs the rule that protein hits are reported as the minimum set that explains all observable peptides. Progenesis QI for Proteomics software does not take a strict parsimonious approach due to the over‐stringency as described previously^51^; however, for the resolution of conflicts, if two proteins contain some common peptides, the protein with fewer peptides is grouped with the protein with a higher number of peptides which are a superset of the subsumed protein’s peptides. The lead protein is the one with the greatest coverage or the highest score when the coverages of two or more proteins are the same. Lead identity peptide data are always used for quantitation, and further details regarding this approach are given on the software website (www.nonlinear.com).

### Statistics

Hierarchical clustering and self-organising maps (SOM) clustering were performed by R programming as described previously^52,53^. Briefly, SOM clustering was performed on data consisting of X number of samples using R package SOM with parameters (.xdim = 5, ydim = 6, topol = “hexa”, neigh = “gaussian”). Data were centred and scaled before performing the clustering. The hclust function in R was used for generating the heatmaps. MetaboAnalyst 4.0 (https://www.metaboanalyst.ca/) was used for generating individual and combined ROC curves and calculating area under the curve (AUC) values^21^. A non-parametric Mann-Whitney test was performed on cases vs. controls and 0.05 was set as the cut-off for p-value. Principal component analysis was performed using Progenesis QI for Proteomics (v4.0). Orthogonal projections for latent structures-discriminant analysis (OPLS-DA) giving S-Plot was generated by the EZInfo 3.0 software with default parameters. Proteomics data were pareto scaled prior to OPLS-DA modelling. The script that was used to calculate Pearson correlation coefficients and p values to demonstrate the correlation between hormone values and protein expression can be found in supplementary information. Calculations were performed in R programming language.

## Supplementary information

Supplementary information file

## Data Availability

The mass spectrometry proteomics data have been deposited into the ProteomeXchange Consortium via the PRIDE^54^ partner repository with the dataset identifiers PXD012034 and 10.6019/PXD012034.

## References

[1] Teede HJ (2018). Recommendations from the international evidence-based guideline for the assessment and management of polycystic ovary syndrome. Hum. Reprod..

[2] Skiba Marina A, Islam Rakibul M, Bell Robin J, Davis Susan R (2018). Understanding variation in prevalence estimates of polycystic ovary syndrome: a systematic review and meta-analysis. Human Reproduction Update.

[3] March WA (2010). The prevalence of polycystic ovary syndrome in a community sample assessed under contrasting diagnostic criteria. Hum. Reprod..

[4] Bozdag G, Mumusoglu S, Zengin D, Karabulut E, Yildiz BO (2016). The prevalence and phenotypic features of polycystic ovary syndrome: a systematic review and meta-analysis. Human Reproduction.

[5] Torchen LC (2017). Cardiometabolic Risk in PCOS: More than a Reproductive Disorder. *Curr*. *Diab Rep*. 137-017.

[6] Gonzalez F (2015). Nutrient-Induced Inflammation in Polycystic Ovary Syndrome: Role in the Development of Metabolic Aberration and Ovarian Dysfunction. Semin. Reprod. Med..

[7] Bahri Khomami M (2019). Increased maternal pregnancy complications in polycystic ovary syndrome appear to be independent of obesity-A systematic review, meta-analysis, and meta-regression. Obes. Rev..

[8] Bahri Khomami M (2019). The role of maternal obesity in infant outcomes in polycystic ovary syndrome-A systematic review, meta-analysis, and meta-regression. Obes. Rev..

[9] Palomba S (2015). Pregnancy complications in women with polycystic ovary syndrome. Hum. Reprod. Update.

[10] Koster MP (2015). Placental characteristics in women with polycystic ovary syndrome. Hum. Reprod..

[11] Palomba S (2013). Macroscopic and microscopic findings of the placenta in women with polycystic ovary syndrome. Hum. Reprod..

[12] Insenser M, Martinez-Garcia MA, Montes R, San-Millan JL, Escobar-Morreale HF (2010). Proteomic analysis of plasma in the polycystic ovary syndrome identifies novel markers involved in iron metabolism, acute-phase response, and inflammation. J. Clin. Endocrinol. Metab..

[13] Ma X (2007). Proteomic analysis of human ovaries from normal and polycystic ovarian syndrome. MHR: Basic science of reproductive medicine.

[14] Ambekar AS (2015). Proteomics of follicular fluid from women with polycystic ovary syndrome suggests molecular defects in follicular development. J. Clin. Endocrinol. Metab..

[15] Cortón M (2008). Proteomic analysis of human omental adipose tissue in the polycystic ovary syndrome using two-dimensional difference gel electrophoresis and mass spectrometry. Human Reproduction.

[16] Borro M (2007). Proteomic analysis of peripheral T lymphocytes, suitable circulating biosensors of strictly related diseases. Clin. Exp. Immunol..

[17] Atiomo WU, Khalid S, Ziauddin A, Tooth D, Layfield R (2009). Framework for a systems approach to proteomic biomarker profiling in polycystic ovary syndrome. Expert Review of Proteomics.

[18] Khan GH, Galazis N, Docheva N, Layfield R, Atiomo W (2015). Overlap of proteomics biomarkers between women with pre-eclampsia and PCOS: a systematic review and biomarker database integration. Hum. Reprod..

[19] Cuevas AM, Germain AM (2011). A Failed Pregnancy Stress Test: A New and Under-Recognized Cardiovascular Risk Factor. Curr. Atheroscler. Rep..

[20] Piltonen TT (2019). Circulating antimullerian hormone and steroid hormone levels remain high in pregnant women with polycystic ovary syndrome at term. Fertil. Steril..

[21] Chong J (2018). MetaboAnalyst 4.0: towards more transparent and integrative metabolomics analysis. Nucleic Acids Res..

[22] Gibson-Helm M, Teede H, Dunaif A, Dokras A (2017). Delayed Diagnosis and a Lack of Information Associated With Dissatisfaction in Women With Polycystic Ovary Syndrome. J. Clin. Endocrinol. Metab..

[23] Huang Chu-Chun, Chou Chia-Hung, Chen Shee-Uan, Ho Hong-Nerng, Yang Yu-Shih, Chen Mei-Jou (2019). Increased platelet factor 4 and aberrant permeability of follicular fluid in PCOS. Journal of the Formosan Medical Association.

[24] Gidwani S (2014). Polycystic ovary syndrome influences the level of serum amyloid A and activity of phospholipid transfer protein in HDL(2) and HDL(3). Hum. Reprod..

[25] Scarinci E (2018). Increased fibulin-1 plasma levels in polycystic ovary syndrome (PCOS) patients: possible contribution to the link between PCOS and cardiovascular risk. J. Endocrinol. Invest..

[26] Kim YS (2013). Apolipoprotein A-IV as a novel gene associated with polycystic ovary syndrome. Int. J. Mol. Med..

[27] Lai Y (2016). Circulating Zinc-alpha2-glycoprotein levels and Insulin Resistance in Polycystic Ovary Syndrome. Sci. Rep..

[28] Liu M (2015). Serum levels of TSP-1, NF-κB and TGF-β1 in polycystic ovarian syndrome (PCOS) patients in northern China suggest PCOS is associated with chronic inflammation. Clin Endocrinol.

[29] Sarig G, Brenner B (2004). Coagulation, inflammation, and pregnancy complications. Lancet.

[30] Bränn E, Edvinsson A, Rostedt Punga A, Sundström-Poromaa I, Skalkidou A (2019). Inflammatory and anti-inflammatory markers in plasma: from late pregnancy to early postpartum. Sci. Rep..

[31] Lacroix M, Kina E, Hivert M (2013). Maternal/Fetal Determinants of Insulin Resistance in Women During Pregnancy and in Offspring Over Life. Current Diabetes Reports.

[32] Ferguson-Smith A, Cattanach BM, Barton SC, Beechey CV, Surani MA (1991). Embryological and molecular investigations of parental imprinting on mouse chromosome 7. Nature.

[33] Constancia M (2002). Placental-specific IGF-II is a major modulator of placental and fetal growth. Nature.

[34] Sibley CP (2004). Placental-specific insulin-like growth factor 2 (Igf2) regulates the diffusional exchange characteristics of the mouse placenta. Proc. Natl. Acad. Sci. USA.

[35] Sferruzzi-Perri A, Owens JA, Pringle KG, Robinson JS, Roberts CT (2006). Maternal Insulin-Like Growth Factors-I and -II Act via Different Pathways to Promote Fetal Growth. endo.

[36] Steegers EAP, von Dadelszen P, Duvekot JJ, Pijnenborg R (2010). Pre-eclampsia. The Lancet.

[37] Blatt AZ, Pathan S, Ferreira VP (2016). Properdin: a tightly regulated critical inflammatory modulator. Immunol. Rev..

[38] Blatt AZ (2016). Properdin-Mediated C5a Production Enhances Stable Binding of Platelets to Granulocytes in Human Whole Blood. J. Immunol..

[39] Snyder ML, Shields KJ, Korytkowski MT, Sutton-Tyrrell K, Talbott EO (2014). Complement protein C3 and coronary artery calcium in middle-aged women with polycystic ovary syndrome and controls. Gynecol. Endocrinol..

[40] Yang S (2011). Serum complement C3 has a stronger association with insulin resistance than high-sensitivity C-reactive protein in women with polycystic ovary syndrome. Fertil. Steril..

[41] Lynch AM, Salmon JE (2010). Dysregulated Complement Activation as a Common Pathway of Injury in Preeclampsia and Other Pregnancy Complications. Placenta.

[42] Girirajan S (2013). Refinement and discovery of new hotspots of copy-number variation associated with autism spectrum disorder. Am. J. Hum. Genet..

[43] Cherskov, A. *et al*. Polycystic ovary syndrome and autism: A test of the prenatal sex steroid theory. *Transl*. *Psychiatry*. **8**, 136-018-0186-7, 10.1038/s41398-018-0186-7 (2018). 10.1038/s41398-018-0186-7 PMC6068102 30065244

[44] Chao J, Bledsoe G, Chao L (2016). Protective Role of Kallistatin in Vascular and Organ Injury. Hypertension (Dallas, Tex.: 1979).

[45] Ceperuelo-Mallafre V (2009). Circulating and adipose tissue gene expression of zinc-alpha2-glycoprotein in obesity: its relationship with adipokine and lipolytic gene markers in subcutaneous and visceral fat. J. Clin. Endocrinol. Metab..

[46] Iliadis S (2015). Corticotropin-releasing hormone and postpartum depression: A longitudinal study. Psychoneuroendocrino.

[47] Kallak TK (2017). Maternal and female fetal testosterone levels are associated with maternal age and gestational weight gain. Eur. J. Endocrinol..

[48] Saraswat M (2017). Human Spermatozoa Quantitative Proteomic Signature Classifies Normo- and Asthenozoospermia. Mol. Cell. Proteomics.

[49] Saraswat M (2017). Comparative proteomic profiling of the serum differentiates pancreatic cancer from chronic pancreatitis. Cancer Medicine.

[50] Silva JC, Gorenstein MV, Li GZ, Vissers JP, Geromanos SJ (2006). Absolute quantification of proteins by LCMSE: a virtue of parallel MS acquisition. Mol. Cell. Proteomics.

[51] Serang O, Moruz L, Hoopmann MR, Kall L (2012). Recognizing uncertainty increases robustness and reproducibility of mass spectrometry-based protein inferences. J. Proteome Res..

[52] Joenvaara S (2018). Quantitative N-glycoproteomics reveals altered glycosylation levels of various plasma proteins in bloodstream infected patients. PLoS One.

[53] Saraswat M, Mäkitie A, Agarwal R, Joenväärä S, Renkonen S (2017). Oral squamous cell carcinoma patients can be differentiated from healthy individuals with label-free serum proteomics. Br. J. Cancer.

[54] Vizcaino JA (2016). 2016 update of the PRIDE database and its related tools. Nucleic Acids Res..

