# Supplementary material for: Thromboinflammatory changes in plasma proteome of pregnant women with PCOS detected by quantitative label-free proteomics
Source: Sci Rep. 2019 Nov 26;9:17578. doi: 10.1038/s41598-019-54067-4 (PMC6879536; doi:10.1038/s41598-019-54067-4)
Supplement: Supplementary file 1 — Supplementary information file [file 41598_2019_54067_MOESM1_ESM.pdf]

Supplementary information file

Thromboinflammatory changes in plasma proteome of pregnant women with PCOS detected by quantitative label-free proteomics

Arffman RK[1,+], Saraswat M[2,3,+], Joenväärä S[2,3], Khatun M[1], Agarwal R[4], Tohmola T[5], Sundström-Poromaa I[6], Renkonen R[2,3] & Piltonen TT[1,*]

[1] Department of Obstetrics and Gynecology, PEDEGO Research Unit, Medical Research Center, Oulu University Hospital, University of Oulu, Oulu, Finland

[2] Transplantation Laboratory, Haartman Institute, University of Helsinki, Helsinki, Finland

[3] HUSLAB, Helsinki University Hospital, Helsinki, Finland

[4] Department of Reproductive biology, All India Institute of Medical Sciences, Ansari Nagar, New Delhi 110029, India

[5] Department of Biosciences, University of Helsinki, Finland

[6] Department of Women's and Children's Health, Uppsala University, Sweden

* terhi.piltonen@oulu.fi

[+] these authors contributed equally to this work.

Protein name	UniProt ID	Mann-Whitney P-value	Peptide Count	Unique Peptides	Confidence Score	Highest Mean Condition	Lowest Mean Condition	Normalized abundances																										
								Control 1	Control 2	Control 3	Control 4	Control 5	Control 6	Control 7	Control 8	Control 9	Control 10	Control 11	Control 12	Control 13	Control 14	Control 15	Control 16	Control 17	Control 18	Control 19	Control 20	PCOS 1	PCOS 2	PCOS 3	PCOS 4	PCOS 5	PCOS 6	PCOS 7
Protein SAA2-SAA4	A0A096LPE2;P35542;P0DJI9	0,00184	11	8	107,1003	Case	Control	75929	96201	72231	86971	162920	103857	68293	133624	137242	100250	91541	53811	86838	44228	101038	56072	69180	114529	111726	131589	256915	148169	219028	208394	107433	107734	116114
POTE ankyrin domain family member F	A5A3E0;Q6S8J3	0,05132	7	4	44,8008	Case	Control	175736	275366	153591	156634	316993	174576	141294	171137	167405	181467	157683	155910	152629	124006	127562	152711	185467	182979	155479	237982	497661	194866	311591	251998	172553	133706	174341
cDNA FLJ55673_ highly similar to Complement factor B	B4E1Z4;P00751	0,08762	141	109	1152,9432	Case	Control	4262344	4852797	3541583	3274096	5727789	6121539	3896000	5841480	6521894	5046898	6334122	3840231	5060160	4053030	3931402	4815475	5334006	3835251	8558668	6142097	8200672	10247138	6777636	5788358	3618047	4355388	5435212
Uncharacterized protein (Fragment)	H0YCG3	0,00219	4	2	21,4915	Control	Case	32523	40706	15170	31403	6186	15841	21333	15656	9867	20616	11791	21243	17216	14860	25670	24948	23164	24202	26425	27337	5389	24747	9860	3786	5885	12594	7596
Uncharacterized protein (Fragment)	H0YJW9	0,08762	16	3	129,5322	Control	Case	71006	85608	34771	34750	34111	37053	29580	25490	25101	24974	29813	32473	38812	26730	39872	38323	47321	42651	70117	25331	42919	43448	18434	29591	24804	31614	18440
Apolipoprotein L1	O14791	0,06406	23	20	173,2402	Control	Case	353156	329015	275758	292931	310216	307258	262150	244074	279170	261989	277504	237251	386093	315068	349566	243876	213671	325715	462705	375220	278701	669385	260408	208928	149623	296004	144630
Disintegrin and metalloproteinase domain-containing protein 12	O43184	0,22755	11	9	65,162	Control	Case	465819	293774	631091	542252	378013	304688	378185	304742	200468	327697	286298	276326	405135	282907	291361	317001	382527	482288	781065	550518	204332	398132	818429	201024	253985	447912	301083
CD5 antigen-like	O43866	0,48897	6	6	45,4362	Control	Case	4583	4366	19139	7817	47657	10759	47143	13232	10652	4767	6637	56063	11474	13130	9793	6804	20766	7065	19083	10280	24571	20313	4914	8514	10173	4662	20513
Ficolin-3	O75636	0,40169	6	4	56,3943	Control	Case	53550	63267	30309	30390	47795	33956	26842	29390	35164	33060	24079	16232	33964	26682	46666	43689	36807	71974	65335	57906	39929	34201	40059	37607	17691	57794	37603
Attractin	O75882	0,24459	32	28	219,5994	Case	Control	600779	428131	461481	568823	1003426	621171	565975	781331	987426	699959	1579473	567247	683635	690516	443090	397816	538731	507035	460885	815343	1685429	732807	1033894	597760	604299	437661	515234
Apolipoprotein M	O95445	0,33908	9	7	58,8403	Case	Control	53366	28887	56860	27539	49118	48656	72263	40851	48947	51590	36926	43719	40547	34700	45107	36577	40060	38779	151203	111619	58075	81550	257165	39393	22599	63501	26613
Ceruloplasmin	P00450	0,35956	219	209	1723,513	Case	Control	19394811	16785251	10567193	10459819	17284931	15278697	14465258	15289899	17111275	11690731	15528222	9955808	16785091	13902500	12241547	13983333	20565458	20888610	20712971	18143711	20672173	28234571	13902230	19334668	10637639	12400174	15350086
Prothrombin	P00734	0,14032	80	71	621,8528	Case	Control	3307196	3379195	2400799	3026875	4385481	3633215	2934636	2876111	3472580	2530749	2779913	2317397	2281433	2331222	3946946	3088057	3632337	4182592	3570538	3312238	3967737	3148589	3314179	3809725	2956505	3314841	3447511
Complement C1r subcomponent	P00736	0,24459	37	34	323,614	Case	Control	541491	537520	452087	436188	881218	753739	477427	664414	740081	544314	727869	420801	504613	442392	666554	508314	593827	592712	530169	572690	1025026	690571	560805	693461	441310	473126	653909
Haptoglobin-related protein	P00739	0,09676	21	5	244,0256	Case	Control	131141	82241	374192	162444	390769	203025	276603	192804	412038	80441	131724	153745	94558	189694	226542	151599	285005	351072	154801	257221	221281	173075	340866	361896	169959	403824	213850
Coagulation factor IX	P00740	0,38045	12	10	80,9906	Case	Control	344164	332108	263912	341628	686103	461943	401765	487810	427065	390082	472856	406391	413830	369915	456550	306003	324718	455260	428037	820307	765136	448680	368094	531207	426591	330011	365085
Coagulation factor X	P00742	0,29958	17	15	100,5396	Case	Control	101461	103983	70484	78035	101037	107170	74658	86901	91740	82531	91070	51329	96907	97415	115155	77078	91214	107903	117385	154316	170371	122792	103490	111690	71452	69567	87909
Plasminogen	P00747;Q15195;Q02325;P35900	0,03191	103	93	923,9142	Case	Control	3925384	2523859	2912211	2659284	6047262	3006489	2956318	3398585	4115746	2296673	2921225	1652653	3604148	2387740	2813559	2552870	3554142	4179813	4160799	3475824	4906051	4335248	4075983	3744728	3118108	3589628	2992726
Coagulation factor XII	P00748	0,00050	15	11	115,4921	Case	Control	172269	271392	101787	72474	170877	198797	149934	230295	244430	182607	86242	172005	138464	136804	168202	191810	204516	175479	171843	129669	267976	246459	231183	186239	276622	374765	285498
Carbonic anhydrase 1	P00915	0,10659	16	14	143,0313	Control	Case	89384	98286	95628	91885	203287	673580	91599	136148	118208	118677	130892	62747	81695	90212	1227967	122235	78335	98641	81657	123613	161667	104669	130522	120184	90129	130647	170694
Carbonic anhydrase 2	P00918	0,42323	8	7	73,102	Control	Case	8974	11114	8135	7997	36611	115192	8814	9637	11858	12087	12068	8284	9331	8100	273695	9094	13373	11878	7205	15581	32630	6449	6067	30027	10341	11941	33719
Antithrombin-III	P01008	0,22755	56	53	525,9525	Control	Case	3069050	1966769	2356662	2472656	3875951	3661139	3489938	3476710	4148037	2836735	4054890	2618608	3639730	3628825	3328312	3368170	3459287	4073769	3806506	3259070	4426712	5808229	2186825	3181959	1978116	2582179	3058236
Alpha-1-antichymotrypsin	P01011	0,48897	68	62	524,746	Case	Control	1499971	1540216	865147	1428689	1559826	2166564	1682255	2030013	1671426	1495397	1413605	1114450	1575257	1512611	2339729	1870840	1789969	2090705	2885108	2334762	2026747	3128466	2986084	1696251	775013	1227593	1408144
Angiotensinogen	P01019	0,44499	85	82	416,1131	Case	Control	6451349	7285232	3908985	5726156	6278233	6934463	6487630	5706734	6664464	5193604	5541571	5654027	6488069	4592566	6615266	6005119	6045796	9021505	12267468	8065771	11094295	11315739	8050499	4511815	4297181	6218323	4248163
Complement C3	P01024;Q96MT0	0,42323	396	364	2707,6239	Case	Control	25461042	26221871	18234650	17428854	16743231	19504821	18207570	18438424	19458421	15661188	17483504	13306197	23440487	16692978	19922642	21349574	23765831	19126276	28537254	24315153	20176069	26813314	24017300	17745595	13484840	24946462	16296356
Complement C5	P01031	0,19556	83	74	711,3527	Control	Case	2299692	2516136	1627634	2067750	2146398	2025212	2194436	2100285	1864506	1915863	1908121	1477699	1932673	1707162	1612685	2062095	2267021	2640041	3568478	3668880	1790671	2970515	3209443	1728782	1496066	1965673	1589748
Cystatin-C	P01034	0,00504	3	2	26,6734	Control	Case	39189	27708	18823	13105	16908	36460	23786	28675	19612	12126	41170	10908	24372	29563	15420	22684	48811	32499	17264	15693	15888	17312	15817	7065	8323	16428	11877
Kininogen-1	P01042	0,22755	55	50	452,3826	Case	Control	2939129	2788387	1999787	2118947	3605850	2449299	1458994	2091845	3125000	2113730	2284789	1745633	2682287	2018802	2287247	2150547	2683977	3040861	2891973	2445511	2375935	2643884	2328286	3405589	1892029	2674730	3157593
Insulin-like growth factor II	P01344	0,00050	2	2	16,3705	Case	Control	26373	23766	36992	24850	23666	18571	18341	32557	27406	24246	67577	46373	93342	38809	15499	30692	18703	45760	57060	97056	157010	98552	104524	124423	90910	44953	82337
Hemoglobin subunit delta	P02042	0,33908	44	36	178,7864	Control	Case	88294	102615	105521	73673	83320	1075362	60774	58520	88590	63282	79419	78567	80152	66875	3913379	251888	61896	68832	83833	139877	86218	76478	89192	70453	78723	99549	223830
Apolipoprotein E	P02649	0,02476	48	45	385,7214	Case	Control	1536411	1517691	1323960	1215615	1508180	999185	1119901	1409043	1797246	1196292	1626934	835182	1153370	1064049	866151	1225076	910668	1130012	1403310	1461828	1474590	1646414	2083258	1343837	1251006	1637140	1216202
Apolipoprotein C-I	P02654	0,05741	15	15	83,975	Case	Control	150075	135814	144387	117847	313023	187068	167287	205069	240232	170447	156085	120463	202121	91569	204928	141984	131460	264697	332452	240259	273339	177065	303541	213212	165204	267753	168383
Apolipoprotein C-II	P02655	0,44499	17	17	131,557	Case	Control	706718	731406	755644	622404	787711	518991	596387	824328	1239528	602918	976381	808105	540023	736607	546859	721849	440173	812380	1602603	1150586	1174241	1856228	879497	582797	565658	559472	467405
Apolipoprotein C-III O	P02656	0,02476	26	23	125,0012	Case	Control	790444	368452	538591	537766	1148240	643131	618936	1112999	1472041	771391	1776234	658477	653183	964680	453187	591449	513662	525433	703310	755891	1449160	834783	938849	951783	1059468	650931	1251350
Band 3 anion transport protein	P02730	0,21120	5	3	32,3846	Control	Case	1602	5131	1311	2648	5013	26058	3608	4686	3382	2458	9887	1016	3895	2834	123803	8827	2391	2805	3833	11524	2789	6178	20381	1577	685	1551	4205
C-reactive protein	P02741	0,29958	2	2	13,6962	Control	Case	14045	35995	11907	9654	8816	28143	6627	17622	9553	12611	17847	13675	24631	14054	15130	17303	17956	11408	11218	24897	17878	15427	25134	12745	8983	10927	8452
Serum amyloid P-component	P02743	0,07914	8	6	57,8421	Control	Case	263286	333628	234637	144676	97455	174344	236512	202971	251900	155678	279222	179621	237032	263257	183111	246394	298712	536775	611891	251945	187457	398782	68673	137142	128030	404820	81359
Complement C1q subcomponent subunit A	P02745	0,22755	7	3	44,6284	Control	Case	106819	57259	24357	32184	62258	99293	81959	96361	73591	50669	56033	30175	87421	84445	51319	70394	57242	134049	64127	50583	34011	139502	74396	41627	35818	41283	66902
Complement C1q subcomponent subunit B	P02746	0,33908	12	8	104,4779	Case	Control	140158	152649	108413	137977	120745	147623	132305	133495	108935	92143	92945	86712	119456	129958	114600	106187	115046	178995	219526	148125	139252	222840	204228	100748	74274	113679	85325
Complement C1q subcomponent subunit C	P02747	0,29958	6	5	40,863	Case	Control	54264	56736	50720	56632	42277	46468	41499	36854	27608	46322	56515	47465	66909	59079	34227	47555	54656	76779	94918	79342	32820	116778	107465	36165	37600	56262	36712
Complement component C9	P02748	0,31907	43	40	386,9734	Case	Control	1624060	2065673	1065160	1519242	2243581	1905828	1447047	1620373	2170306	1339232	1525948	1332546	1551260	1292103	1435856	1596001	1579781	1785995	1893818	2380973	2371646	2134811	2044773	1809659	1008685	1405099	1568714
Beta-2-glycoprotein 1	P02749	0,42323	56	53	337,5775	Case	Control	2732725	2994987	1346520	2049067	3583538	4364995	2969798	3092340	3585525	2391739	3355791	2391045	2941399	2632833	2740174	2828463	3747465	4484946	3621139	2127321	3523495	4061572	2708320	3088365	2863097	2122164	3092648
Leucine-rich alpha-2-glycoprotein	P02750	0,42323	28	26	296,4767	Case	Control	1057887	1258293	728726	898758	1377762	1846044	1171073	1243747	1430305	847564	1252071	1156034	1092910	1055138	1148104	1708032	1325623	1704825	1113417	1971954	2544669	1679185	989734	1715578	536679	1028605	1094452
Retinol-binding protein 4	P02753	0,42323	18	17	215,8521	Control	Case	654776	590558	286889	398519	780017	609785	659440	757416	944574	572046	645387	415451	535697	485339	497202	493848	515343	575323	738377	527285	578152	755436	418416	649928	371169	486544	698678
Protein AMBP	P02760	0,19556	42	39	322,0821	Case	Control	1552772	1674975	1009287	1249135	1621013	1903005	1169266	1355552	1347669	1392846	1485896	1022964	1543598	1360903	1321975	1102219	1489134	1617029	1374788	1089903	1592023	2032420	1648306	1562578	1115184	1211551	1331965
Alpha-2-HS-glycoprotein	P02765	0,46693	69	63	397,743	Case	Control	7184124	5663782	4287867	4928503	6246122	5968078	6086535	5182029	5865764	5365175	4801123	3621799	6019370	4144406	4507721	5529048	5571827	5338024	12332143	10769496	5384866	11395805	11483175	4838342	3536756	6040904	3816683
Transthyretin	P02766	0,06406	17	16	186,4477	Case	Control	419900	642943	199547	257714	340525	436798	343415	273936	269461	240257	188971	278139	398629	237972	197292	274877	671474	790410	334781	672069	451999	572988	500166	425919	180647	685020	503805
Vitamin D-binding protein	P02774;Q5VZM2;Q7L523	0,22755	173	151	1108,3413	Case	Control	11227760	11995390	6862804	9133932	11230259	11259551	11272701	10735274	12788161	8675647	11820823	9385872	10610679	9541119	9141103	11099048	11700537	12365782	14609150	12157124	14070719	15601440	10510706	12708508	9106639	8592422	12447463
Platelet basic protein	P02775	0,00935	8	7	68,7062	Case	Control	168689	103289	135036	110494	293896	176452	91465	124796	81215	99205	69908	60038	110518	74177	125205	72057	105837	144588	116363	270366	100445	248879	162607	277022	291780	204357	165717
Platelet factor 4	P02776;P10720	0,00219	6	6	78,7425	Case	Control	110088	53452	70522	57433	392135	150955	75801	89681	64198	75687	40292	26487	65321	55138	108899	30691	64631	114176	57045	224963	114841	217306	80264	329082	267748	155042	188551
Lactotransferrin	P02788	0,28064	4	3	24,2674	Control	Case	90857	10366	44712	39354	69282	28897	75830	80518	78459	58691	94000	134690	73050	130618	30098	94008	82116	54775	27934	71548	74091	45212	45790	52860	68999	87696	57096
Hemopexin	P02790;Q2M389;Q5VXU9;Q8N987;Q9NZ08	0,01443	122	111	739,7604	Case	Control	7073532	6458409	3925855	5862616	9458435	7437960	8182955	7693208	10326557	6153931	6559364	5729398	7737685	6404103	5806491	7596292	7653429	9897508	9474773	7845735	12391479	10772370	7986402	9564489	5889376	8075555	9868921
Plasma kallikrein	P03952	0,40169	35	29	308,3202	Case	Control	756926	631148	408811	427220	611315	601811	590028	584289	607729	465542	517284	332106	532168	395963	495151	501398	551381	734555	1131805	489629	749968	997384	508239	527682	398271	607297	484449
C4b-binding protein alpha chain	P04003	0,22755	34	32	315,8209	Case	Control	998589	952507	674542	920267	923331	630237	612098	823979	905621	792866	619729	633067	696974	707399	587368	673673	872833	1126587	1177330	807393	1101493	1063068	1024231	969578	599710	861024	567152
Vitronectin	P04004	0,03191	79	62	456,8093	Case	Control	3232627	3477242	1797311	2103136	3786344	3321728	2626897	2712579	3486829	2328318	3529109	2392690	3260896	2813345	2526772	2742270	2990667	2619091	2520103	2409449	3824208	3859622	2785093	3699153	2247621	2878913	3804801
Catalase O	P04040	0,48897	3	3	20,0093	Control	Case	2186	4730	1185	906	2864	14837	2197	1809	2332	2301	2580	676	1153	2675	65848	4269	1321	804	750	2745	1631	950	858	5027	1234	2859	4595
Vitamin K-dependent protein C	P04070	0,06406	3	2	18,6941	Control	Case	13181	15511	5167	5422	3922	3989	7450	7817	6233	4661	6605	4088	9660	4796	10499	8953	10305	21596	8850	3488	800	9603	6206	2199	2239	15679	3873
Annexin A1	P04083	0,44499	15	10	87,9993	Case	Control	590616	333377	475117	437071	778473	582197	425798	589470	675691	351839	700360	568473	437956	622797	310866	544346	497131	377053	316457	624787	783677	772315	463262	549128	402332	331064	467592
Phosphatidylcholine-sterol acyltransferase	P04180	0,05132	15	11	104,2323	Control	Case	159225	125750	160353	134849	114319	184420	100926	124667	126271	113660	147537	96751	150294	105942	278383	115057	144193	135700	119791	214635	85744	143569	180033	84313	101240	124760	99798
Histidine-rich glycoprotein	P04196	0,35956	24	23	167,5128	Case	Control	431422	354276	376650	203822	383917	610540	229731	492061	396364	376197	265135	347359	649602	459022	429128	450688	666447	649499	517952	493158	192827	645736	786767	307227	269535	640467	268567
Alpha-1B-glycoprotein	P04217	0,02172	94	83	638,9677	Case	Control	6025482	5112611	3790340	4846970	7884486	8941814	5464667	5795237	5190036	5539242	7904961	5543259	6796854	5563365	4572236	5187761	6971427	6309045	4175468	7461501	10290218	9004785	8711255	7888842	5424124	5248309	7451117
Keratin_ type II cytoskeletal 1	P04264	0,11711	3	2	26,1691	Control	Case	7002	58344	5114	7190	14806	5105	3886	10475	10886	3541	5725	7392	4785	6045	25676	4307	6255	4664	33249	16134	12518	11149	8143	10301	6732	7178	13006
Sex hormone-binding globulin	P04278	0,21120	31	27	284,3138	Case	Control	1552505	1289492	676927	828208	1604465	1112633	1256810	834646	1312335	1102970	955039	1028247	1052955	758220	861643	918482	980708	973343	1837467	1239265	1343751	1428815	1683794	1531349	503992	816424	1190280
Glyceraldehyde-3-phosphate dehydrogenase	P04406	0,04575	4	4	30,3497	Control	Case	6929	6987	9637	11908	5813	32118	6758	10165	11474	7760	11370	7858	9610	12954	68635	9349	6283	6611	5513	11763	6601	12348	7031	5252	7331	5387	6878
HLA class II histocompatibility antigen_ DP beta 1 chain	P04440	0,29958	4	3	24,551	Control	Case	98454	253000	41298	39496	31697	58708	61233	38321	67390	43599	40139	58547	58483	43152	149806	388310	104822	67899	108094	83507	57174	188700	45161	24477	60151	67203	47386
Apolipoprotein D	P05090	0,21120	16	14	116,2265	Case	Control	308085	157229	223005	278039	374461	253486	279466	225368	333324	230073	181946	262023	296311	229679	921732	190486	196621	266811	274592	494755	329649	327874	558946	507694	190068	214247	259452
Plasma protease C1 inhibitor	P05155	0,05132	37	34	330,8414	Control	Case	921900	892488	1005576	1070902	1135155	845224	842994	851379	1729109	1021558	1249065	731244	811122	847528	1036391	1159335	807971	899775	924458	1233162	601911	890833	922211	1361434	344088	813867	752853
Complement factor I	P05156	0,44499	36	31	329,4776	Case	Control	898129	898960	876595	882644	932728	1129449	812083	1001798	1055166	793384	1040117	774724	938988	890273	858553	833292	1142513	958296	940903	1368240	1197053	1765092	1343404	967210	742376	748378	775888
Tetranectin	P05452	0,07130	11	9	89,5476	Control	Case	77659	248568	40470	62944	112040	130047	112165	86993	95726	75378	82901	70635	80828	67343	76836	67290	92164	112561	121340	71672	96366	92591	56709	82075	38660	69481	62285
Thyroxine-binding globulin	P05543	0,29958	22	21	201,6064	Control	Case	555274	723322	304502	429056	369073	490205	475869	415180	490380	448306	439326	356832	541412	443503	400663	471892	541280	621198	700708	374635	376840	794049	431774	463094	275717	539335	406084
Heparin cofactor 2	P05546	0,18064	47	38	444,8945	Control	Case	1567689	1506746	691642	990347	786850	904618	1069513	1205246	1007426	1010847	1022585	940670	1298034	807820	905696	833315	1317885	1509598	1951609	1489736	855395	1643196	1489767	743103	598622	1282878	630687
Cholinesterase	P06276	0,38045	9	9	55,1826	Case	Control	325975	322680	277464	210501	140388	134257	188951	197242	152245	191117	124159	249869	240325	247894	203336	277453	197219	301851	256426	186837	144664	396436	267539	119706	145048	404451	151070
Complement C2	P06681	0,42323	38	13	311,1739	Case	Control	107983	135107	67360	88274	156637	107179	82195	88722	92590	87170	109157	71992	84961	69452	85484	95722	116973	139441	119676	116855	191885	101277	79319	133042	73618	90729	98640
Apolipoprotein A-IV	P06727	0,02476	56	54	503,5325	Case	Control	1340281	941189	959441	1067715	2099558	1255058	1377331	1090598	1977519	1136120	973779	980115	1390025	1238093	1577378	1210641	1115502	1488601	1386077	2250444	2247352	2096073	1370938	2573939	1069605	1746251	1409427
Vitamin K-dependent protein S	P07225	0,09676	30	25	205,472	Case	Control	677435	581078	647805	558121	654742	522832	448499	620667	639205	523805	619060	596879	581532	573042	571483	511846	579492	761841	762850	703545	738825	902819	840929	743475	498343	675745	454512
Complement component C8 alpha chain	P07357	0,04575	29	25	228,7875	Control	Case	495631	367495	346345	417796	471871	506469	534448	441821	456110	440708	497823	448763	518620	646747	338566	492819	585712	473745	520576	435478	383679	599756	484971	377859	345581	312674	363379
Complement component C8 beta chain	P07358	0,03607	30	25	256,3513	Control	Case	813867	725301	428695	501204	528078	757561	691197	724682	696965	623651	674701	484689	644755	621958	691908	688991	692785	749475	1128562	594833	674369	1056825	625284	398108	351236	502038	414508
Complement component C8 gamma chain	P07360	0,12835	12	11	101,0484	Control	Case	306462	325877	274266	286900	358879	383888	253003	363020	350296	296747	265028	255088	233107	269387	266831	293318	395059	426125	345969	267770	305702	316918	285349	341697	225800	265924	250133
Thrombospondin-1	P07996	0,00691	9	6	46,5519	Case	Control	36656	41112	22783	23276	56801	35342	40505	30755	55416	28395	39368	36193	38256	37435	38953	46694	38918	33362	74078	47737	75655	111373	37582	60182	44647	42955	49538
Corticosteroid-binding globulin	P08185	0,07914	18	14	192,7942	Control	Case	1342872	841827	639762	756177	545691	654025	788501	443043	429536	690275	489673	463149	787660	663374	808396	739948	826037	949318	1600880	1657526	466687	842549	1134475	365238	379367	677660	312934
Monocyte differentiation antigen CD14	P08571	0,18064	10	7	71,9078	Control	Case	41364	61365	33183	43384	27872	236304	40768	36011	35494	39268	30589	35280	52683	38605	251457	46726	38412	82556	76918	22230	31389	68211	13146	30108	22868	58574	75579
Complement factor H	P08603	0,11711	89	73	840,1667	Case	Control	2319455	1134795	1969468	1581348	1919310	1984696	1497373	2293334	2278403	1868381	2066801	1505532	1864110	1600173	1616794	1583639	2250154	2114290	1788077	1940273	2636577	2587982	2266565	1936789	1425971	2141869	1691507
Alpha-2-antiplasmin	P08697	0,24459	34	33	322,5676	Control	Case	1306492	677830	797915	939221	1452482	1112591	1339193	919746	1390128	1041710	1170805	763010	1133718	1002172	1054437	1340745	1047449	1374403	2129998	1333061	1049928	2061630	947158	1117800	631969	1222576	861725
Complement C1s subcomponent	P09871	0,35956	31	30	271,3573	Case	Control	660408	763613	495803	673136	557691	527471	658914	601159	549260	613525	636893	497735	501978	556999	364959	510117	534727	719192	858781	822031	718342	994200	838072	453703	453218	513901	434535
Complement C4-A	P0C0L4	0,16646	209	57	1880,3779	Control	Case	693889	358492	202256	286917	247676	303982	257274	191836	420103	298138	332634	263108	266666	240144	297222	434822	285805	551329	298558	339027	147320	376371	337589	250156	182648	363782	237583
Complement C4-B	P0C0L5	0,31907	157	8	1941,9655	Control	Case	438786	218391	132133	248658	163080	137153	215922	138513	156301	228070	189194	201102	198950	249399	267384	241682	286504	429525	216664	240088	108551	274717	331309	165944	187391	258075	98128
Serum amyloid A-1 protein	P0DJI8	0,22755	6	4	51,7825	Case	Control	13300	13838	14972	13834	20253	19360	11400	19061	16063	13335	16063	9857	14365	10621	20522	8672	16709	9447	11652	25401	53964	14995	26805	17478	11142	9118	14974
Chorionic somatomammotropin hormone 1	P0DML2;P0DML3;P01241	0,06406	17	14	135,3467	Case	Control	302295	208620	242095	264173	607770	326856	239345	357229	509274	306829	390881	271719	236488	361319	286445	231516	221059	320542	270729	368083	652523	395273	356487	528341	245637	231516	472923
Complement component C7	P10643	0,46693	37	32	316,6553	Control	Case	583836	605937	537600	586101	863502	660217	500718	569168	704751	482434	416232	439732	545977	539157	490816	458969	563605	731295	524551	752733	656520	673331	604154	742589	391662	454322	460159
Clusterin	P10909	0,00128	41	34	355,9224	Case	Control	1084859	1135910	828406	939016	1361873	890818	832539	1037829	1374200	863453	934321	841371	1092781	866719	1003323	965403	1108179	1147736	1377328	1218782	1698691	1534137	1601220	1522106	1085581	1183450	1255770
Mannose-binding protein C	P11226	0,21120	5	4	38,1388	Control	Case	318378	395468	277476	304091	368436	387028	245494	300990	290033	299744	309766	231410	368203	264483	398763	230214	299451	395530	397912	476562	286800	381230	245721	285706	330105	311004	284747
Pregnancy-specific beta-1-glycoprotein 1	P11464	0,24459	45	25	292,5922	Control	Case	645178	391499	665503	498685	545737	651709	423668	627734	1198034	518229	441497	313989	265488	159076	309730	473448	244961	379772	413839	291216	372681	195282	126882	742376	379266	342963	793609
Pregnancy-specific beta-1-glycoprotein 2	P11465	0,09676	16	11	113,4191	Case	Control	45393	31277	42441	64357	98799	134636	138759	115670	175802	165106	66062	115103	19049	247767	41799	40453	123396	50597	24750	85806	458911	81399	156315	157717	86550	30612	141196
Complement component C6	P13671	0,42323	46	38	387,9291	Control	Case	712674	920492	709578	621312	1241223	954263	848754	957432	1074912	599750	863008	505330	719964	779023	653307	721829	887260	1072624	1131882	838998	1268415	1084682	602530	904648	572704	758385	673486
Bone marrow proteoglycan	P13727	0,26230	15	10	123,7038	Control	Case	235833	184983	208896	272274	358813	307324	261195	312358	333412	211639	247843	248497	245221	235942	254068	192221	225418	268549	176365	166876	237535	215820	234776	211538	174319	299768	263624
Carboxypeptidase N catalytic chain	P15169	0,24459	11	10	89,9379	Control	Case	143299	146025	113822	118316	146395	119203	143627	125922	153243	126277	107412	126444	143568	158573	161600	140746	173044	156825	139679	178556	80417	201269	146377	136955	84990	160645	104536
Insulin-like growth factor-binding protein 3	P17936	0,24459	5	5	34,0461	Case	Control	57892	39359	66005	44920	59887	50050	34179	37108	41201	40569	37136	48589	40533	47299	35102	26289	79956	54897	38567	53928	70011	43907	46622	58974	40420	52148	38002
Lipopolysaccharide-binding protein	P18428	0,29958	9	8	70,5103	Case	Control	366038	224685	251687	220082	181181	242732	233375	214594	150939	193099	231524	258538	315989	222263	154102	289031	357496	283378	402074	238593	170269	481052	350934	103196	209602	402855	334226
Inter-alpha-trypsin inhibitor heavy chain H2	P19823	0,00364	94	82	711,1136	Case	Control	3314732	3512553	2374506	2561871	3802670	3473968	3364508	3472970	3591874	2622568	3468775	2214449	2840824	3181834	2840041	2857076	3410133	3586500	4523849	3578709	4566488	5683062	3896213	4640286	2652470	3596460	3665936
Inter-alpha-trypsin inhibitor heavy chain H1	P19827	0,03191	79	70	676,8673	Case	Control	3403919	3518601	2405359	2445056	3467255	4658608	3396201	3474436	3328405	2805068	3753970	2476122	3284785	3042914	3050468	2935602	3852253	4590951	5465899	4580046	4798795	6124611	3339270	4722849	3237035	3705346	3926777
Pregnancy zone protein	P20742	0,46693	125	101	1067,8661	Control	Case	1058239	3437619	2738690	3769604	3629608	3567567	1103968	3295647	7799462	4458770	3798360	2044686	1328299	1748095	6104713	8148054	1309909	1746140	1000611	5568733	4404806	1419073	4399821	2633076	4204501	2954833	1588476
C4b-binding protein beta chain	P20851	0,11711	7	7	63,9588	Control	Case	129613	69294	104885	115255	114401	81055	97819	125288	143805	88131	133374	105127	87601	132058	79122	95763	139190	167081	134469	90099	135368	148139	85021	92841	68827	85648	66979
Glutathione peroxidase 3	P22352	0,06406	7	6	41,337	Case	Control	133481	188733	164655	128787	222633	195919	125788	192531	240339	198436	257062	220393	288772	184238	296079	168748	161967	247428	229024	462706	347934	231269	406428	372965	225488	159224	218569
Carboxypeptidase N subunit 2	P22792	0,07130	19	16	236,772	Control	Case	852547	833149	653254	1013066	465415	483911	885960	726359	770169	850713	882283	1017373	752279	776222	921143	874771	1055739	1181703	1501447	1598360	440731	2257726	833361	372983	522413	892317	266774
Fibulin-1	P23142	0,00691	24	19	190,5288	Case	Control	231124	108337	235858	247005	389181	110288	126000	295254	167408	212532	124893	143101	174823	284155	164213	103116	210772	140389	103032	161288	448564	228119	359181	266118	182398	243134	256849
Zinc-alpha-2-glycoprotein	P25311	0,04068	18	16	123,6621	Control	Case	348800	398140	218569	277301	307133	239814	304378	196347	215041	250836	247308	185110	325108	312898	143736	247852	240313	337647	268115	182112	221027	251949	127454	252524	113360	228625	202208
Hepatocyte growth factor-like protein	P26927	0,46693	2	2	12,6484	Case	Control	2636	7373	3601	607	1097	2318	2258	2672	3209	3068	1043	515	3509	2147	1055	1204	2097	5147	3664	3555	723	3309	5601	1527	2005	6931	1766
Serum paraoxonase/arylesterase 1	P27169	0,02814	27	23	296,9113	Control	Case	1904217	1356843	693968	879959	1643764	1175861	1741638	2021632	1351861	1147322	1349593	1224766	1531696	1142462	803612	1243980	1200084	1804892	2210509	985735	1028889	2076199	1097869	581536	880394	1092969	734351
Embryonic growth/differentiation factor 1	P27539	0,02172	2	2	4,8324	Control	Case	20443	7553	19372	9297	13646	46460	11281	4821	8852	9246	16458	18634	14536	14953	416045	25941	21332	15309	6462	10752	3374	21316	8158	3237	9063	13534	6657
Properdin	P27918	0,00006	4	3	30,1267	Case	Control	4359	1625	3661	3035	12845	3588	8076	4448	5234	6239	23134	10949	34282	6451	717	11368	2571	15760	14302	24785	216714	93170	216942	114246	114519	68238	93953
Kallistatin	P29622	0,18064	15	13	124,3326	Control	Case	450451	499592	294879	396806	358032	424427	253032	371036	278979	338313	331740	264058	411498	268940	397650	278166	327247	381646	380562	292381	352549	408405	492071	254222	187681	323182	201823
Flavin reductase (NADPH)	P30043	0,31907	2	2	12,4365	Case	Control	4080	985	1891	5262	11332	13298	4902	9487	8104	10735	13163	3812	2324	5554	42142	4792	9447	5741	1207	9563	20074	8173	6561	18860	8791	1986	1931
Peroxiredoxin-2	P32119;Q06830	0,46693	14	14	110,0415	Case	Control	25190	40132	26430	24439	36720	270030	32507	31131	27872	36741	35180	16516	26742	24937	672584	52631	29921	25247	19359	39747	41657	28205	21692	28157	22399	38969	62522
Keratin type I cytoskeletal 9	P35527	0,38045	3	3	18,6235	Control	Case	3853	63874	4955	3749	3612	2281	1810	2736	4515	1630	1510	1864	4475	1661	4805	6178	6471	3569	22374	6862	2337	6493	4104	3497	1727	6033	1912
Insulin-like growth factor-binding protein complex acid labile subunit	P35858	0,09676	32	31	359,363	Control	Case	886364	1571600	665888	651957	824913	757879	659783	610307	722795	601165	567917	530468	636403	706648	820737	685867	738918	836950	1025950	637851	617731	738141	522175	700683	400236	979535	594006
Pigment epithelium-derived factor	P36955	0,10659	24	20	193,8052	Control	Case	330617	358627	311522	387992	326707	434422	363101	356785	257547	293889	246973	290821	302150	337377	512017	232707	386125	474293	450089	356373	627592	497795	222428	268438	209540	300236	213277
Complement factor H-related protein 2	P36980	0,48897	8	4	52,5115	Case	Control	45092	26513	16036	40023	64530	53155	22437	51756	48806	39726	21278	28816	45050	27793	122297	24564	43938	40197	36216	46550	92634	51559	32692	62719	23890	20332	32161
Prostaglandin-H2 D-isomerase	P41222	0,00429	3	2	16,8754	Control	Case	7248	10190	7757	7287	9441	7878	6947	4774	8636	6765	4010	5718	6998	5849	5091	8511	5353	9391	7427	9404	3276	4665	3793	4220	2342	7772	6839
Phosphatidylinositol 4,5-bisphosphate 3-kinase catalytic subunit alpha isoform	P42336	0,10659	7	3	33,0155	Control	Case	21570	35611	24504	21216	9999	12265	11904	11556	7233	12359	7833	16745	18391	9114	13414	12447	13576	30650	23976	12939	3302	9808	20030	10047	9427	33111	10445
Biotinidase	P43251	0,05741	11	10	74,5339	Control	Case	352852	246972	398328	224360	263181	207790	255597	293382	264370	250869	351694	268194	224080	221247	243115	228244	291944	289636	315615	249411	211689	260053	162849	213733	302285	273896	152650
Afamin	P43652	0,12835	70	63	598,5427	Case	Control	2649331	2502550	1831811	1822095	2558303	2305397	2177872	2760532	2609142	2027006	2490858	1859813	2261740	2452843	2051851	1980929	2453410	2676763	3089104	2187075	3228542	3409909	2412716	2672525	1901400	2710982	1919061
Mannan-binding lectin serine protease 1	P48740	0,48897	15	11	88,1646	Control	Case	194663	286771	192440	134051	113199	146387	108397	101151	96808	131111	93118	83118	146088	137403	266120	101244	191097	207900	192282	216660	130709	122971	207191	141220	129363	221624	110709
Selenoprotein P	P49908	0,15302	4	3	30,3431	Case	Control	187540	81690	135664	134404	211837	158680	138571	148672	206213	148058	170309	133709	235633	127561	183182	101403	164108	144969	241366	237374	375344	267482	264903	198569	104407	119488	152026
Granzyme M	P51124	0,00219	2	2	16,0762	Control	Case	225364	234042	140768	322125	23418	64389	167347	112964	38829	106579	126993	242073	196604	93618	36036	217282	144280	234869	405192	303642	18871	218555	4822	2192	15125	100215	6666
Lumican	P51884	0,48897	22	16	183,0766	Case	Control	273100	357984	265621	218081	412108	391159	280372	182067	502212	220315	286993	306849	266086	268785	230594	234969	283104	375654	407305	306355	572389	434167	192163	483238	132540	270196	234323
Biliverdin reductase A	P53004	0,06406	2	2	11,628	Control	Case	578065	185019	218309	313862	402562	690680	650397	802573	892642	525258	618842	84307	497675	972619	718237	473758	677276	392089	481712	345530	334367	1262098	387125	400967	343310	260921	63010
Apolipoprotein C-IV	P55056	0,02476	9	8	75,8587	Case	Control	68844	86795	88634	67392	85706	86166	55595	85057	85184	74248	73128	50594	103663	52478	276008	62408	56134	98452	161367	72324	155551	88708	126497	83846	80208	122948	91811
Actin_ cytoplasmic 1	P60709;P63261	0,00691	12	3	97,7242	Case	Control	707	3507	911	624	5108	2684	1334	403	2864	1377	604	865	1240	316	2124	2339	2377	917	2077	8017	2242	4325	1556	2726	16427	13615	3119
Beta-2-microglobulin	P61769	0,21120	4	3	28,8068	Control	Case	30095	29703	10569	16026	11802	19738	16127	20053	26745	15233	19916	24558	28091	64213	13295	11930	22865	18762	17938	14289	16648	49674	11307	15745	8334	21322	16323
Hemoglobin subunit beta	P68871;P02100	0,48897	46	37	320,3852	Control	Case	254691	316527	173373	163190	466659	12748280	240622	274413	272715	356334	363468	149902	232187	185063	22767249	1525041	243968	252411	218475	299699	291557	262071	184673	294861	150540	320783	4318508
Hemoglobin subunit gamma-1	P69891;P69892	0,35956	3	2	40,3195	Control	Case	401	576	307	221	275	15018	356	39	118	460	421	219	1348	3145	52508	1759	230	2822	1030	4000	82	685	285	203	551	1141	7776
Hemoglobin subunit alpha	P69905	0,44499	47	44	217,4591	Control	Case	210966	282209	125132	137378	328017	8366810	173161	190852	275320	287373	337079	148740	216905	236837	30233049	1873630	209889	222115	175353	359380	319826	278641	196470	301064	110585	226149	1662729
Phosphatidylinositol-glycan-specific phospholipase D	P80108	0,05132	17	14	134,8355	Case	Control	157718	141875	108383	132159	336671	119112	134630	139280	239562	134592	119682	149166	177739	120822	123146	128442	124780	192002	142844	172660	214213	143309	145469	276560	142371	129126	208215
Pregnancy-specific beta-1-glycoprotein 9	Q00887	0,16646	27	15	248,382	Case	Control	189835	131353	246394	189220	[illegible]	[illegible]	[illegible]	[illegible]	[illegible]	[illegible]	[illegible]	[illegible]	[illegible]	[illegible]	[illegible]	[illegible]	[illegible]	[illegible]	[illegible]	[illegible]	[illegible]	[illegible]	[illegible]	[illegible]	[illegible]	[illegible]	[illegible]
Complement factor H-related protein 1	Q03591	[illegible]	[illegible]	[illegible]	[illegible]	[illegible]	[illegible]	[illegible]	[illegible]	[illegible]	[illegible]	[illegible]	[illegible]	[illegible]	[illegible]	[illegible]	[illegible]	[illegible]	[illegible]	[illegible]	[illegible]	[illegible]	[illegible]	[illegible]	[illegible]	[illegible]	[illegible]	[illegible]	[illegible]	[illegible]	[illegible]	[illegible]	[illegible]	[illegible]	[illegible]	[illegible]
Inter-alpha-trypsin inhibitor heavy chain H3	Q06033	[illegible]	[illegible]	[illegible]	[illegible]	[illegible]	[illegible]	[illegible]	[illegible]	[illegible]	[illegible]	[illegible]	[illegible]	[illegible]	[illegible]	[illegible]	[illegible]	[illegible]	[illegible]	[illegible]	[illegible]	[illegible]	[illegible]	[illegible]	[illegible]	[illegible]	[illegible]	[illegible]	[illegible]	[illegible]	[illegible]	[illegible]	[illegible]	[illegible]	[illegible]	[illegible]
Galectin-3-binding protein	Q08380	[illegible]	[illegible]	[illegible]	[illegible]	[illegible]	[illegible]	[illegible]	[illegible]	[illegible]	[illegible]	[illegible]	[illegible]	[illegible]	[illegible]	[illegible]	[illegible]	[illegible]	[illegible]	[illegible]	[illegible]	[illegible]	[illegible]	[illegible]	[illegible]	[illegible]	[illegible]	[illegible]	[illegible]	[illegible]	[illegible]	[illegible]	[illegible]	[illegible]	[illegible]	[illegible]
ADP-ribosyl cyclase/cyclic ADP-ribose hydrolase 2	Q10588	[illegible]	[illegible]	[illegible]	[illegible]	[illegible]	[illegible]	[illegible]	[illegible]	[illegible]	[illegible]	[illegible]	[illegible]	[illegible]	[illegible]	[illegible]	[illegible]	[illegible]	[illegible]	[illegible]	[illegible]	[illegible]	[illegible]	[illegible]	[illegible]	[illegible]	[illegible]	[illegible]	[illegible]	[illegible]	[illegible]	[illegible]	[illegible]	[illegible]	[illegible]	[illegible]
EGF-containing fibulin-like extracellular matrix protein 1	Q12805	[illegible]	[illegible]	[illegible]	[illegible]	[illegible]	[illegible]	[illegible]	[illegible]	[illegible]	[illegible]	[illegible]	[illegible]	[illegible]	[illegible]	[illegible]	[illegible]	[illegible]	[illegible]	[illegible]	[illegible]	[illegible]	[illegible]	[illegible]	[illegible]	[illegible]	[illegible]	[illegible]	[illegible]	[illegible]	[illegible]	[illegible]	[illegible]	[illegible]	[illegible]	[illegible]
Putative pregnancy-specific beta-1-glycoprotein 7	Q13046	[illegible]	[illegible]	[illegible]	[illegible]	[illegible]	[illegible]	[illegible]	[illegible]	[illegible]	[illegible]	[illegible]	[illegible]	[illegible]	[illegible]	[illegible]	[illegible]	[illegible]	[illegible]	[illegible]	[illegible]	[illegible]	[illegible]	[illegible]	[illegible]	[illegible]	[illegible]	[illegible]	[illegible]	[illegible]	[illegible]	[illegible]	[illegible]	[illegible]	[illegible]	[illegible]
Pappalysin-1	Q13219	[illegible]	[illegible]	[illegible]	[illegible]	[illegible]	[illegible]	[illegible]	[illegible]	[illegible]	[illegible]	[illegible]	[illegible]	[illegible]	[illegible]	[illegible]	[illegible]	[illegible]	[illegible]	[illegible]	[illegible]	[illegible]	[illegible]	[illegible]	[illegible]	[illegible]	[illegible]	[illegible]	[illegible]	[illegible]	[illegible]	[illegible]	[illegible]	[illegible]	[illegible]	[illegible]
Apolipoprotein F	Q13790	[illegible]	[illegible]	[illegible]	[illegible]	[illegible]	[illegible]	[illegible]	[illegible]	[illegible]	[illegible]	[illegible]	[illegible]	[illegible]	[illegible]	[illegible]	[illegible]	[illegible]	[illegible]	[illegible]	[illegible]	[illegible]	[illegible]	[illegible]	[illegible]	[illegible]	[illegible]	[illegible]	[illegible]	[illegible]	[illegible]	[illegible]	[illegible]	[illegible]	[illegible]	[illegible]
Ectonucleotide pyrophosphatase/phosphodiesterase family member 2	Q13822	[illegible]	[illegible]	[illegible]	[illegible]	[illegible]	[illegible]	[illegible]	[illegible]	[illegible]	[illegible]	[illegible]	[illegible]	[illegible]	[illegible]	[illegible]	[illegible]	[illegible]	[illegible]	[illegible]	[illegible]	[illegible]	[illegible]	[illegible]	[illegible]	[illegible]	[illegible]	[illegible]	[illegible]	[illegible]	[illegible]	[illegible]	[illegible]	[illegible]	[illegible]	[illegible]
[illegible]	[illegible]	[illegible]	[illegible]	[illegible]	[illegible]	[illegible]	[illegible]	[illegible]	[illegible]	[illegible]	[illegible]	[illegible]	[illegible]	[illegible]	[illegible]	[illegible]	[illegible]	[illegible]	[illegible]	[illegible]	[illegible]	[illegible]	[illegible]	[illegible]	[illegible]	[illegible]	[illegible]	[illegible]	[illegible]	[illegible]	[illegible]	[illegible]	[illegible]	[illegible]
Hyaluronan-binding protein 2	Q14520	[illegible]	[illegible]	[illegible]	[illegible]	[illegible]	[illegible]	[illegible]	[illegible]	[illegible]	[illegible]	[illegible]	[illegible]	[illegible]	[illegible]	[illegible]	[illegible]	[illegible]	[illegible]	[illegible]	[illegible]	[illegible]	[illegible]	[illegible]	[illegible]	[illegible]	[illegible]	[illegible]	[illegible]	[illegible]	[illegible]	[illegible]	[illegible]	[illegible]	[illegible]	[illegible]
Inter-alpha-trypsin inhibitor heavy chain H4	Q14624	[illegible]	[illegible]	[illegible]	[illegible]	[illegible]	[illegible]	[illegible]	[illegible]	[illegible]	[illegible]	[illegible]	[illegible]	[illegible]	[illegible]	[illegible]	[illegible]	[illegible]	[illegible]	[illegible]	[illegible]	[illegible]	[illegible]	[illegible]	[illegible]	[illegible]	[illegible]	[illegible]	[illegible]	[illegible]	[illegible]	[illegible]	[illegible]	[illegible]	[illegible]	[illegible]
Pregnancy-specific beta-1-glycoprotein 5	Q15238	[illegible]	[illegible]	[illegible]	[illegible]	[illegible]	[illegible]	[illegible]	[illegible]	[illegible]	[illegible]	[illegible]	[illegible]	[illegible]	[illegible]	[illegible]	[illegible]	[illegible]	[illegible]	[illegible]	[illegible]	[illegible]	[illegible]	[illegible]	[illegible]	[illegible]	[illegible]	[illegible]	[illegible]	[illegible]	[illegible]	[illegible]	[illegible]	[illegible]	[illegible]	[illegible]
[illegible]	[illegible]	[illegible]	[illegible]	[illegible]	[illegible]	[illegible]	[illegible]	[illegible]	[illegible]	[illegible]	[illegible]	[illegible]	[illegible]	[illegible]	[illegible]	[illegible]	[illegible]	[illegible]	[illegible]	[illegible]	[illegible]	[illegible]	[illegible]	[illegible]	[illegible]	[illegible]	[illegible]	[illegible]	[illegible]	[illegible]	[illegible]	[illegible]	[illegible]	[illegible]
Extracellular matrix protein 1	Q16610	[illegible]	[illegible]	[illegible]	[illegible]	[illegible]	[illegible]	[illegible]	[illegible]	[illegible]	[illegible]	[illegible]	[illegible]	[illegible]	[illegible]	[illegible]	[illegible]	[illegible]	[illegible]	[illegible]	[illegible]	[illegible]	[illegible]	[illegible]	[illegible]	[illegible]	[illegible]	[illegible]	[illegible]	[illegible]	[illegible]	[illegible]	[illegible]	[illegible]	[illegible]	[illegible]
LINE-1 type transposase domain-containing protein 1	Q5T7N2	[illegible]	[illegible]	[illegible]	[illegible]	[illegible]	[illegible]	[illegible]	[illegible]	[illegible]	[illegible]	[illegible]	[illegible]	[illegible]	[illegible]	[illegible]	[illegible]	[illegible]	[illegible]	[illegible]	[illegible]	[illegible]	[illegible]	[illegible]	[illegible]	[illegible]	[illegible]	[illegible]	[illegible]	[illegible]	[illegible]	[illegible]	[illegible]	[illegible]	[illegible]	[illegible]
Tubulin polyglutamylase TTLL7	Q6ZT98	[illegible]	[illegible]	[illegible]	[illegible]	[illegible]	[illegible]	[illegible]	[illegible]	[illegible]	[illegible]	[illegible]	[illegible]	[illegible]	[illegible]	[illegible]	[illegible]	[illegible]	[illegible]	[illegible]	[illegible]	[illegible]	[illegible]	[illegible]	[illegible]	[illegible]	[illegible]	[illegible]	[illegible]	[illegible]	[illegible]	[illegible]	[illegible]	[illegible]	[illegible]	[illegible]
Collectin-11	Q9BWP8	[illegible]	[illegible]	[illegible]	[illegible]	[illegible]	[illegible]	[illegible]	[illegible]	[illegible]	[illegible]	[illegible]	[illegible]	[illegible]	[illegible]	[illegible]	[illegible]	[illegible]	[illegible]	[illegible]	[illegible]	[illegible]	[illegible]	[illegible]	[illegible]	[illegible]	[illegible]	[illegible]	[illegible]	[illegible]	[illegible]	[illegible]	[illegible]	[illegible]	[illegible]	[illegible]
Dedicator of cytokinesis protein 4	Q8N1I0	[illegible]	[illegible]	[illegible]	[illegible]	[illegible]	[illegible]	[illegible]	[illegible]	[illegible]	[illegible]	[illegible]	[illegible]	[illegible]	[illegible]	[illegible]	[illegible]	[illegible]	[illegible]	[illegible]	[illegible]	[illegible]	[illegible]	[illegible]	[illegible]	[illegible]	[illegible]	[illegible]	[illegible]	[illegible]	[illegible]	[illegible]	[illegible]	[illegible]	[illegible]	[illegible]
F-box/LRR-repeat protein 6	Q8N531	[illegible]	[illegible]	[illegible]	[illegible]	[illegible]	[illegible]	[illegible]	[illegible]	[illegible]	[illegible]	[illegible]	[illegible]	[illegible]	[illegible]	[illegible]	[illegible]	[illegible]	[illegible]	[illegible]	[illegible]	[illegible]	[illegible]	[illegible]	[illegible]	[illegible]	[illegible]	[illegible]	[illegible]	[illegible]	[illegible]	[illegible]	[illegible]	[illegible]	[illegible]	[illegible]
Inactive dipeptidyl peptidase 10	Q8N608	[illegible]	[illegible]	[illegible]	[illegible]	[illegible]	[illegible]	[illegible]	[illegible]	[illegible]	[illegible]	[illegible]	[illegible]	[illegible]	[illegible]	[illegible]	[illegible]	[illegible]	[illegible]	[illegible]	[illegible]	[illegible]	[illegible]	[illegible]	[illegible]	[illegible]	[illegible]	[illegible]	[illegible]	[illegible]	[illegible]	[illegible]	[illegible]	[illegible]	[illegible]	[illegible]
Complement factor H-related protein 5	Q9BXR6	[illegible]	[illegible]	[illegible]	[illegible]	[illegible]	[illegible]	[illegible]	[illegible]	[illegible]	[illegible]	[illegible]	[illegible]	[illegible]	[illegible]	[illegible]	[illegible]	[illegible]	[illegible]	[illegible]	[illegible]	[illegible]	[illegible]	[illegible]	[illegible]	[illegible]	[illegible]	[illegible]	[illegible]	[illegible]	[illegible]	[illegible]	[illegible]	[illegible]	[illegible]	[illegible]
Proteoglycan 4	Q92954	[illegible]	[illegible]	[illegible]	[illegible]	[illegible]	[illegible]	[illegible]	[illegible]	[illegible]	[illegible]	[illegible]	[illegible]	[illegible]	[illegible]	[illegible]	[illegible]	[illegible]	[illegible]	[illegible]	[illegible]	[illegible]	[illegible]	[illegible]	[illegible]	[illegible]	[illegible]	[illegible]	[illegible]	[illegible]	[illegible]	[illegible]	[illegible]	[illegible]	[illegible]	[illegible]
Carboxypeptidase B2	Q96IY4	[illegible]	[illegible]	[illegible]	[illegible]	[illegible]	[illegible]	[illegible]	[illegible]	[illegible]	[illegible]	[illegible]	[illegible]	[illegible]	[illegible]	[illegible]	[illegible]	[illegible]	[illegible]	[illegible]	[illegible]	[illegible]	[illegible]	[illegible]	[illegible]	[illegible]	[illegible]	[illegible]	[illegible]	[illegible]	[illegible]	[illegible]	[illegible]	[illegible]	[illegible]	[illegible]
Beta-Ala-His dipeptidase	Q96KN2	[illegible]	[illegible]	[illegible]	[illegible]	[illegible]	[illegible]	[illegible]	[illegible]	[illegible]	[illegible]	[illegible]	[illegible]	[illegible]	[illegible]	[illegible]	[illegible]	[illegible]	[illegible]	[illegible]	[illegible]	[illegible]	[illegible]	[illegible]	[illegible]	[illegible]	[illegible]	[illegible]	[illegible]	[illegible]	[illegible]	[illegible]	[illegible]	[illegible]	[illegible]	[illegible]
N-acetylmuramoyl-L-alanine amidase	Q96PD5	[illegible]	[illegible]	[illegible]	[illegible]	[illegible]	[illegible]	[illegible]	[illegible]	[illegible]	[illegible]	[illegible]	[illegible]	[illegible]	[illegible]	[illegible]	[illegible]	[illegible]	[illegible]	[illegible]	[illegible]	[illegible]	[illegible]	[illegible]	[illegible]	[illegible]	[illegible]	[illegible]	[illegible]	[illegible]	[illegible]	[illegible]	[illegible]	[illegible]	[illegible]	[illegible]
[illegible]	[illegible]	[illegible]	[illegible]	[illegible]	[illegible]	[illegible]	[illegible]	[illegible]	[illegible]	[illegible]	[illegible]	[illegible]	[illegible]	[illegible]	[illegible]	[illegible]	[illegible]	[illegible]	[illegible]	[illegible]	[illegible]	[illegible]	[illegible]	[illegible]	[illegible]	[illegible]	[illegible]	[illegible]	[illegible]	[illegible]	[illegible]	[illegible]	[illegible]	[illegible]
Fetuin-B	Q9UGM5	[illegible]	[illegible]	[illegible]	[illegible]	[illegible]	[illegible]	[illegible]	[illegible]	[illegible]	[illegible]	[illegible]	[illegible]	[illegible]	[illegible]	[illegible]	[illegible]	[illegible]	[illegible]	[illegible]	[illegible]	[illegible]	[illegible]	[illegible]	[illegible]	[illegible]	[illegible]	[illegible]	[illegible]	[illegible]	[illegible]	[illegible]	[illegible]	[illegible]	[illegible]	[illegible]
Leucyl-cystinyl aminopeptidase	Q9UIQ6	[illegible]	[illegible]	[illegible]	[illegible]	[illegible]	[illegible]	[illegible]	[illegible]	[illegible]	[illegible]	[illegible]	[illegible]	[illegible]	[illegible]	[illegible]	[illegible]	[illegible]	[illegible]	[illegible]	[illegible]	[illegible]	[illegible]	[illegible]	[illegible]	[illegible]	[illegible]	[illegible]	[illegible]	[illegible]	[illegible]	[illegible]	[illegible]	[illegible]	[illegible]	[illegible]
Protein Z-dependent protease inhibitor	Q9UK55	[illegible]	[illegible]	[illegible]	[illegible]	[illegible]	[illegible]	[illegible]	[illegible]	[illegible]	[illegible]	[illegible]	[illegible]	[illegible]	[illegible]	[illegible]	[illegible]	[illegible]	[illegible]	[illegible]	[illegible]	[illegible]	[illegible]	[illegible]	[illegible]	[illegible]	[illegible]	[illegible]	[illegible]	[illegible]	[illegible]	[illegible]	[illegible]	[illegible]	[illegible]	[illegible]
Pregnancy-specific beta-1-glycoprotein 11	Q9UQ72	[illegible]	[illegible]	[illegible]	[illegible]	[illegible]	[illegible]	[illegible]	[illegible]	[illegible]	[illegible]	[illegible]	[illegible]	[illegible]	[illegible]	[illegible]	[illegible]	[illegible]	[illegible]	[illegible]	[illegible]	[illegible]	[illegible]	[illegible]	[illegible]	[illegible]	[illegible]	[illegible]	[illegible]	[illegible]	[illegible]	[illegible]	[illegible]	[illegible]	[illegible]	[illegible]
Fanconi-associated nuclease 1	Q9Y2M0	[illegible]	[illegible]	[illegible]	[illegible]	[illegible]	[illegible]	[illegible]	[illegible]	[illegible]	[illegible]	[illegible]	[illegible]	[illegible]	[illegible]	[illegible]	[illegible]	[illegible]	[illegible]	[illegible]	[illegible]	[illegible]	[illegible]	[illegible]	[illegible]	[illegible]	[illegible]	[illegible]	[illegible]	[illegible]	[illegible]	[illegible]	[illegible]	[illegible]	[illegible]	[illegible]
Protein FAM65B	Q9Y4F9	[illegible]	[illegible]	[illegible]	[illegible]	[illegible]	[illegible]	[illegible]	[illegible]	[illegible]	[illegible]	[illegible]	[illegible]	[illegible]	[illegible]	[illegible]	[illegible]	[illegible]	[illegible]	[illegible]	[illegible]	[illegible]	[illegible]	[illegible]	[illegible]	[illegible]	[illegible]	[illegible]	[illegible]	[illegible]	[illegible]	[illegible]	[illegible]	[illegible]	[illegible]	[illegible]

Supplementary Table 1. Normalized protein abundances for each woman.

Entrez Gene Name	Symbol	Expression Fold Change	Expression p-value	Location	Family	GenPept/UniProt/ Swiss-Prot Accession	Entrez Gene ID for Human
alpha-1-B glycoprotein	A1BG	1,2961	0,02171846	Extracellular Space	other	P04217	1
apolipoprotein A4	APOA4	1,3313	0,024756011	Extracellular Space	transporter	P06727	337
apolipoprotein C3	APOC3	1,3072	0,024756011	Extracellular Space	transporter	P02656	345
apolipoprotein C4	APOC4	1,1963	0,024756011	Extracellular Space	transporter	P55056	346
apolipoprotein E	APOE	1,2030	0,024756011	Extracellular Space	transporter	P02649	348
complement C8 alpha chain	C8A	-1,1518	0,045752264	Extracellular Space	other	P07357	731
complement C8 beta chain	C8B	-1,1715	0,03607497	Extracellular Space	other	P07358	732
complement factor H related 5	CFHR5	-1,8042	0,028142174	Extracellular Space	other	Q9BXR6	81494
clusterin	CLU	1,3505	0,001283227	Cytoplasm	other	P10909	1191
	Collagen(s)			Extracellular Space	complex		
carboxypeptidase B2	CPB2	-1,2070	0,016579994	Extracellular Space	peptidase	Q96IY4	1361
	ERK1/2			Cytoplasm	group		
coagulation factor XII	F12	1,5844	0,000497325	Extracellular Space	peptidase	P00748	2161
	Fibrinogen			Plasma Membrane	complex		
	Growth hormone			Extracellular Space	group		
	HDL			Plasma Membrane	complex		
	HDL-cholesterol			Other	complex		
hemopexin	HPX	1,2522	0,014427232	Extracellular Space	transporter	P02790	3263
insulin like growth factor 2	IGF2	2,6155	0,000497325	Extracellular Space	growth factor	P01344	3481
	IL1			Extracellular Space	group		
	Iti			Other	complex		
inter-alpha-trypsin inhibitor heavy chain 1	ITIH1	1,2197	0,031905422	Extracellular Space	other	P19827	3697
inter-alpha-trypsin inhibitor heavy chain 2	ITIH2	1,2695	0,003643625	Extracellular Space	other	P19823	3698
	Kallikrein			Extracellular Space	group		
	LDL			Plasma Membrane	complex		
	LDL-cholesterol			Other	complex		
	MAC			Plasma Membrane	complex		
	NCOR-LXR-Oxysterol-RXR-9 cis RA			Nucleus	complex		
	Nr1h			Nucleus	group		
platelet factor 4	PF4	2,0052	0,002190025	Extracellular Space	cytokine	P02776	5196
plasminogen	PLG	1,1738	0,031905422	Extracellular Space	peptidase	P00747	5340
paraoxonase 1	PON1	-1,2807	0,028142174	Extracellular Space	phosphatase	P27169	5444
thrombospondin 1	THBS1	1,5031	0,006906099	Extracellular Space	other	P07996	7057
	VLDL-cholesterol			Other	complex		
vitronectin	VTN	1,1645	0,031905422	Extracellular Space	other	P04004	7448

Supplementary Table 2. All the proteins in the top network affected by PCOS. For the proteins that were differentially expressed in the dataset fold-change is also presented. Only proteins passing the cut-off of 0.05 for Mann-Whitney were utilized.

Entrez Gene Name	Symbol	Expression Fold Change	Expression p-value	Location	Family	GenPept/UniProt/ Swiss-Prot Accession	Entrez Gene ID for Human
actin beta	ACTB	3,112793035	0,006906099	Cytoplasm	other	P60709	60
cyclase associated actin cytoskeleton regulatory protein 2	CAP2			Plasma Membrane	other		10486
C-C motif chemokine receptor 10	CCR10			Plasma Membrane	G-protein coupled receptor		2826
CD59 molecule (CD59 blood group)	CD59			Plasma Membrane	other		966
cell division cycle 42	CDC42			Cytoplasm	enzyme		998
complement factor B	CFB			Extracellular Space	peptidase		629
complement factor H related 4	CFHR4	1,347647495	0,040680565	Extracellular Space	transporter	Q92496	10877
complement factor properdin	CFP	13,28188797	6,01978E-05	Extracellular Space	other	P27918	5199
	coagulation factor			Plasma Membrane	group		
coactosin like F-actin binding protein 1	COTL1			Cytoplasm	other		23406
carboxypeptidase B2	CPB2	-1,206968068	0,016579994	Extracellular Space	peptidase	Q96IY4	1361
cytochrome b5 reductase 3	Cyb5r3			Cytoplasm	enzyme		
ectodermal-neural cortex 1	ENC1			Nucleus	peptidase		8507
F11 receptor	F11R			Plasma Membrane	other		50848
coagulation factor II, thrombin	F2	1,079903228	0,140315596	Extracellular Space	peptidase	P00734	2147
F-box and leucine rich repeat protein 6	FBXL6	1,817499818	0,003643625	Other	enzyme	Q8N531	26233
	FSH			Plasma Membrane	complex		
growth differentiation factor 1	GDF1	-3,810643037	0,02171846	Extracellular Space	growth factor	P27539	2657
	GNRH			Cytoplasm	group		
hepatocyte nuclear factor 4 alpha	HNF4A			Nucleus	transcription regulator		3172
interleukin 1 beta	IL1B			Extracellular Space	cytokine		3553
mitogen-activated protein kinase 8	Jnk			Cytoplasm	group		
microtubule associated protein 7	MAP7	1,362069101	0,02171846	Cytoplasm	other	Q14244	9053
metallothionein 3	Mt3			Cytoplasm	other		
natural cytotoxicity triggering receptor 1	NCR1			Plasma Membrane	transmembrane receptor		9437
NUS1, dehydrodolichyl diphosphate synthase subunit	NUS1			Cytoplasm	enzyme		116150
purinergic receptor P2X 7	P2rx7			Plasma Membrane	ion channel		
	Pka			Cytoplasm	complex		
pro-platelet basic protein	PPBP	1,636079574	0,009350096	Extracellular Space	cytokine	P02775	5473
proline and arginine rich end leucine rich repeat protein	PRELP			Extracellular Space	other		5549
glutaminyl-tRNA synthase (glutamine-hydrolyzing)-like 1	QRSL1			Cytoplasm	enzyme		55278
SAA2-SAA4 readthrough	SAA2-SAA4	1,751834132	0,001837806	Extracellular Space	transporter	A0A096LPE2	100528017
semaphorin 6B	SEMA6B			Plasma Membrane	other		10501
solute carrier family 11 member 1	SLC11A1			Plasma Membrane	transporter		6556
transglutaminase 2	TGM2			Cytoplasm	enzyme		7052

Supplementary Table 3. All the proteins in the second significant network affected by PCOS. For the proteins that were differentially expressed in the dataset, fold-change is also presented. Only proteins passing the cut-off of 0.05 for the Mann-Whitney were utilised.

UniProt ID	Description	Red: 2	Green: 3	Blue: 5	Cyan: 10	Pink: 20	Yellow: 36
P27918	Properdin (CFP)				■	■	■
P60709;P63261	Actin_ cytoplasmic 1 (ACTB)	■	■	■	■	■	■
P01344	Insulin-like growth factor II (IGF2)						■
P02776;P10720	Platelet factor 4 (PF4)					■	■
Q8N531	F-box/LRR-repeat protein 6 (FBXL6)					■	■
A0A096LPE2;P35542;P0DJI9	Protein SAA2-SAA4 (SAA2-SAA4)						■
P02775	Platelet basic protein (PPBP)					■	■
P00748	Coagulation factor XII (F12)	■	■	■	■	■	■
P23142	Fibulin-1 (FBLN1)						■
P07996	Thrombospondin-1 (THBS1)				■	■	■
Q14244	Ensconsin (MAP7)						■
P10909	Clusterin (CLU)					■	■
Q92496	Complement factor H-related protein 4 (CFHR4)				■	■	■
P06727	Apolipoprotein A-IV (APOA4)						■
P02656	Apolipoprotein C-III (APOC3)						■
P04217	Alpha-1B-glycoprotein (A1BG)						■
P19823	Inter-alpha-trypsin inhibitor heavy chain H2 (ITIH2)					■	■
P02790;Q2M389;Q5VXU9;Q8N987;Q9NZ08	Hemopexin (HPX)					■	■
P19827	Inter-alpha-trypsin inhibitor heavy chain H1 (ITIH1)						■
P02649	Apolipoprotein E (APOE)					■	■
P55056	Apolipoprotein C-IV (APOC4)						■
P00747;Q15195;Q02325;P35900	Plasminogen (PLG)						■
P04004	Vitronectin (VTN)					■	■
P07357	Complement component C8 alpha chain (C8A)						■
P07358	Complement component C8 beta chain (C8B)						■
Q96IY4	Carboxypeptidase B2 (CPB2)					■	■
P27169	Serum paraoxonase/ arylesterase 1 (PON1)						■
P25311	Zinc-alpha-2-glycoprotein (AZGP1)					■	■
P41222	Prostaglandin-H2 D-isomerase (PTGDS)			■	■	■	■
P04406	Glyceraldehyde-3-phosphate dehydrogenase (GAPDH)						■
Q9BXR6	Complement factor H-related protein 5 (CFHR5)				■	■	■
H0YCG3	Uncharacterized protein (Fragment)						■
P01034	Cystatin-C (CST3)				■	■	■
P51124	Granzyme M (GZMM)			■	■	■	■
P27539	Embryonic growth/ differentiation factor 1 (GDF1)						■
P03971	Anti-Müllerian hormone (AMH)		■	■	■	■	■

Supplementary Table 4. Proteins included in the calculation of combination ROC-curves presented in Fig 5a.

Rscript:

```
library(xlsx)
clinical =  read.xlsx("datasetname.xlsx", sheetIndex = 1)
expdata  = read.xlsx("datasetname.xlsx", sheetIndex = 2)
clinical= as.data.frame(clinical)
clinical= clinical[,-2]
rownames(clinical)=clinical[,1]
clinical= clinical[,-1]
head(expdata)
expdata = as.data.frame(expdata)
acc=expdata[,1]
rownames(expdata) =acc
colnames(expdata)
expdata1=t(expdata)
expdata1=expdata1[-1,]
dim(expdata1)
expdata1[is.na(expdata1)]=0
expdata2 <- apply(expdata1, 2,as.numeric)
rownames(expdata2) = rownames(expdata1)
dim(expdata2)
dim(clinical)
library(psych)
for (i in 1:8)
{pp = corr.test(expdata2, as.matrix(clinical[,i]), use = "complete", method = "pearson", adjust = "none", alpha = .05 )
r <-pp$r  ## correlation matrix
p <-pp$p ## Raw p-value
rp = cbind(r,p)
colnames(rp) = c("correlation matrix", "p-value")
write.csv(rp, paste0(colnames(clinical)[i]," correlation test_raw_pval",".csv"))}
```

Supplementary information. The script that was used to calculate Pearson correlation coefficients and p values.
